# LRRK2 regulates retrograde synaptic compensation at the *Drosophila* neuromuscular junction

**DOI:** 10.1038/ncomms12188

**Published:** 2016-07-19

**Authors:** Jay Penney, Kazuya Tsurudome, Edward H. Liao, Grant Kauwe, Lindsay Gray, Akiko Yanagiya, Mario R. Calderon, Nahum Sonenberg, A. Pejmun Haghighi

**Affiliations:** 1 Department of Physiology, McGill University, 3655 Promenade Sir William Osler, Montreal, Quebec, Canada H3G 1Y6; 2 Buck Institute for Research on Aging, 8001 Redwood Boulevard, Novato, California 94945, USA; 3 Department of Biochemistry, Goodman Cancer Research Centre, McGill University, McGill University, 1160 Pine Avenue West, Room 614, Montreal, Quebec, Canada H3A 1A3

## Abstract

Parkinson's disease gene leucine-rich repeat kinase 2 (LRRK2) has been implicated in a number of processes including the regulation of mitochondrial function, autophagy and endocytic dynamics; nevertheless, we know little about its potential role in the regulation of synaptic plasticity. Here we demonstrate that postsynaptic knockdown of the fly homologue of LRRK2 thwarts retrograde, homeostatic synaptic compensation at the larval neuromuscular junction. Conversely, postsynaptic overexpression of either the fly or human LRRK2 transgene induces a retrograde enhancement of presynaptic neurotransmitter release by increasing the size of the release ready pool of vesicles. We show that LRRK2 promotes cap-dependent translation and identify Furin 1 as its translational target, which is required for the synaptic function of LRRK2. As the regulation of synaptic homeostasis plays a fundamental role in ensuring normal and stable synaptic function, our findings suggest that aberrant function of LRRK2 may lead to destabilization of neural circuits.

## Abstract (web summary)

Mutations in the protein LRRK2 have been associated with Parkinson's disease but little is still known about the basic functions of the protein in the brain. Here the authors show that in fruit flies, LRRK2 regulates retrograde homeostatic synaptic compensation at the larval neuromuscular junction.

## Graphical abstract

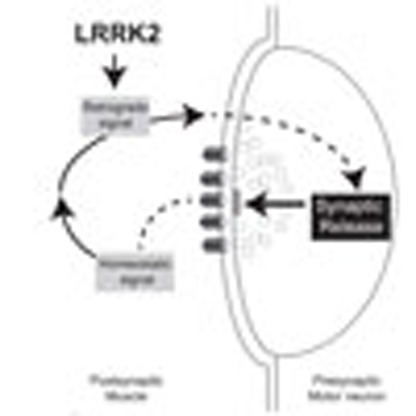

Homeostatic mechanisms ensure stability in neural circuits by adjusting synaptic strength within an optimal range in response to perturbations in synaptic activity^1^,^2^. Synaptic strength at the *Drosophila* larval neuromuscular junction (NMJ) is in part regulated through a homeostatic compensatory mechanism. Genetic manipulations that reduce postsynaptic receptor activity in response to unitary release of transmitter trigger a retrograde signalling cascade that results in a compensatory enhancement in presynaptic neurotransmitter release^3^. The details of the molecular steps that lead to triggering this compensatory synaptic enhancement remain unclear; nevertheless, postsynaptic cap-dependent translational mechanisms, under the control of target of rapamycin (TOR), are critical for maintaining this signalling during larval development^4^. TOR activity is necessary for the normal compensatory retrograde synaptic enhancement and its overexpression in postsynaptic muscles is sufficient for enhancing synaptic strength^4^.

In the past decade, mutations in leucine-rich repeat kinase 2 (LRRK2) have emerged as the most frequently detected and most highly associative mutations causing familial Parkinson's disease^5^,^6^. LRRK2 is a large protein of 2,527 amino acids with multiple functional domains including an ankyrin repeat region, a leucine-rich repeat domain, a kinase domain, a RAS domain, a Ras-of-complex–GTPase domain and a WD40 motif among others^7^. Many mutations in LRRK2 in patients with familial Parkinson's disease are thought to act as gain-of-function mutations^8^. Interestingly, LRRK2 has been implicated in the regulation of translation through interaction with translation initiation factors^9^ and via regulation of ribosomal function^10^. LRRK2 was initially reported to influence protein synthesis through phosphorylation of eukaryotic translation initiation factor 4E (eIF4E)-binding protein (4E-BP), the inhibitor of initiation factor 4E^9^. Later studies, however, have challenged this interaction^10^,^11^. More recently, Martin *et al*.^10^ have suggested that LRRK2 directly phosphorylates the ribosomal protein S15 and thereby enhances protein synthesis. Based on the strong link between translational mechanisms and synaptic homeostasis at the *Drosophila* larval NMJ, we set out to investigate the role of LRRK2 in the regulation of synaptic compensation at the NMJ.

All messenger RNAs in eukaryotes that are transcribed in the nucleus contain a 7-methylguanosine-containing cap structure (m7GpppN) at their 5′-end. The cap facilitates the binding of mRNA and the ribosomal 40S subunit through its direct interaction with eIF4F^12^,^13^, a protein complex that is primarily composed of the three eukaryotic initiation factors eIF4E, eIF4A and eIF4G. The rate-limiting step in the initiation of translation is the binding of eIF4E to the cap. A major regulation of translation is exerted by 4E-BPs, which interfere with the eIF4E–eIF4G interaction and thereby inhibit translation initiation. On binding of eIF4E to the cap structure, eIF4A with its helicase activity unwinds the secondary structure of the 5′-untranslated region (UTR), setting the stage for the binding of the ribosome and start of translation^13^,^14^.

Our findings indicate that loss or knockdown of LRRK2 disrupts the ability of the synapse to undergo retrograde synaptic compensation. In particular, we show that endogenous *Drosophila* dLRRK in postsynaptic muscles, but not in motoneurons, is responsible for maintaining the normal homeostatic compensation at the NMJ. Furthermore, we find that postsynaptic overexpression of either the fly (dLRRK) or the human (hLRRK2) LRRK2 transgenes can induce a retrograde enhancement of presynaptic release, which is fully reversed by limiting protein translation either genetically or pharmacologically. Our findings uncover a function for dLRRK in the regulation of synaptic function and suggest that aberrant function of LRRK2 might lead to dysregulation of synaptic function in neural circuits.

## Results

### LRRK2 promotes cap-dependent translation

Using metabolic [^35^S]-methionine/cysteine labelling, we monitored total protein synthesis in HeLa cells and found that overexpression of wild-type hLRRK2 enhanced total protein synthesis by ∼45% (Supplementary Fig. 1a). Consistently, knockdown of hLRRK2 in HEK293FT cells, which have higher basal LRRK2 levels compared with HeLa cells (Supplementary Fig. 1c,d), led to a decrease of ∼40% in total protein synthesis (Supplementary Fig. 1b). We did not detect any changes in the level of translation initiation factors in response to gain or loss of LRRK2 (Supplementary Fig. 1c,d), precluding the possibility that LRRK2-related changes in translation were due to significant alterations in the expression of these proteins. We then conducted *in vitro* reporter assays to determine the influence of LRRK2 on cap-dependent versus cap-independent translation. Martin *et al*.^10^ have recently reported that hLRRK2 enhanced both cap-dependent and cap-independent translation. Similarly, we found that cap-dependent translation was efficiently enhanced in response to hLRRK2 overexpression; however, our results indicated that hLRRK2 had only a minimal effect on cap-independent translation, which relies on an internal ribosome entry site (IRES) (Supplementary Fig. 1e). We have previously reported that postsynaptic cap-dependent translation mechanisms play a critical role in the regulation of synaptic homeostasis^4^. Therefore, identification of a role for LRRK2 as a promoter of cap-dependent translation prompted us to investigate whether dLRRK exerts any influence on the regulation of synaptic homeostasis at the NMJ.

### dLRRK is required for homeostatic synaptic compensation

**Figure 1:**
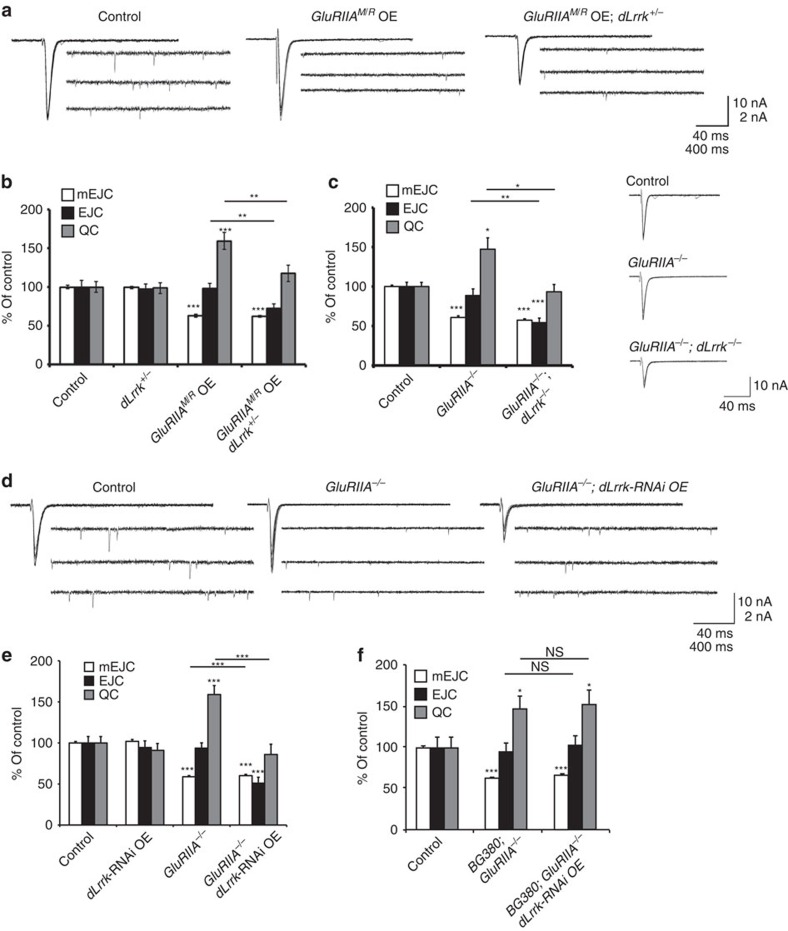
Postsynaptic dLRRK is required for homeostatic synaptic compensation at the *Drosophila* larval NMJ. (**a**) Representative traces of mEJCs and EJCs from Control (*MHC-Gal4*/+), GluRIIA^M/R^ OE (muscle overexpression of UAS*-GluRIIA*^*M/R*^: UAS*-GluRIIA*^*M/R*^ /+; *MHC-Gal4*/+), GluRIIA^M/R^ OE; *dLrrk* heterozygous mutants (UAS*-GluRIIA*^*M/R*^*/+; MHC-Gal4/dLrrk*^*e03680*^). (**b**) Quantification of mEJC, EJC and QC from the genotypes shown in **a** and *dLrrk* heterozygotes. *n*=21, 27, 19 and 19. (**c**) Quantification of mEJC, EJC and QC from Control (*w1118*), *GluRIIA*^*−/−*^ (*GluRIIA*^*SP16*^/*Df(2L)cl-h4*) and *GluRIIA^−/−^*; *dLrrk ^−/−^* (*GluRIIA*^*SP16*^/*Df(2L)cl-h4*; *dLrrk*^*e03680*^), and representative EJC traces. *n*=32, 20 and 16. (**d**) Representative traces of mEJCs and EJCs from control (*24B-Gal4*/+), *GluRIIA* mutants carrying the 24B-Gal4 muscle driver (*Df(2L)cl-h4/ GluRIIA*^*SP16*^*; 24B-Gal4/+*) and *GluRIIA* mutants expressing *dLrrk*-RNAi in the muscle (*Df(2L)cl-h4/ GluRIIA*^*SP16*^*; 24B-Gal4*/UAS*-dLRRK*-RNAi). UAS-*Dcr-2* is also present in all combinations, except for control. (**e**) Quantification of mEJC, EJC and QC from the genotypes shown in **d** and in *dLrrk*-RNAi OE (UAS-*Dcr-2*/+; *24B-Gal4*/UAS-*dLRRK*-RNAi). *n*=19, 20, 18 and 19. (**f**) Quantification of mEJC, EJC and QC from BG380 (*BG380-Gal4*/+; *+/GluRIIA*^*SP16*^), *BG380; GluRIIA^−/−^* (*BG380-Gal4*/+; *Df(2L)cl-h4/ GluRIIA*^*SP16*^) and *BG380; GluRIIA^−/−^*; *dLRRK*-RNAi (*BG380-Gal4*/+; *Df(2L)cl-h4/ GluRIIA*^*SP16*^; +/UAS-*dLRRK*-RNAi). UAS-*Dcr-2* is present in all combinations. *n*=17, 18 and 20. Error bars represent s.e.m. *P<0.05, **P<0.01 and ***P<0.001. See Supplementary Table 1.

Postsynaptic receptors at the *Drosophila* larval NMJ are formed by tetrameric coassembly of glutamate receptor (GluR) subunits A, C, D and E or B, C, D and E^15^,^16^,^17^. Genetic removal of GluRIIA subunit or overexpression of a dominant-negative GluRIIA mutant transgene (UAS-GluRIIA^M/R^) in muscles reduces glutamate-induced synaptic conductance and triggers a retrograde compensatory enhancement in presynaptic release probability^3^. We have recently demonstrated that this synaptic enhancement is critically dependent on the normal activity of postsynaptic cap-dependent translation^4^. Therefore, we set out to test whether loss of *dLrrk* would have a detrimental effect on the normal ability of the NMJ to undergo retrograde compensation. When UAS-GluRIIA^M/R^ is overexpressed in postsynaptic muscles (using MHC-Gal4 driver), miniature excitatory junctional currents (mEJCs) are reduced in amplitude, but EJCs are maintained at wild-type levels, revealing a homeostatic synaptic enhancement in quantal content (QC) (Fig. 1a,b)^18^. We found that heterozygosity for *dLrrk* was sufficient to cause a strong block in homeostatic synaptic enhancement in larvae overexpressing UAS-GluRIIA^M/R^ (mean±s.e.m. for QC was 55.56±3.83 versus 41.07±3.60 for *GluRIIA^M/R^*/+; *MHC-Gal4*/+ versus *GluRIIA^M/R^*/+; *MHC-Gal4*/*dLrrk e03680*, *n*=19 for each genotype and *P*<0.01, one-way analysis of variance) (Fig. 1a,b). At the same time, we measured baseline electrophysiological properties in larvae heterozygous for *dLrrk* and found that they were indistinguishable from control larvae (Fig. 1b). In support of these results, we found that *GluRIIA*; *dLrrk* double mutant larvae were deficient in their ability to exhibit homeostatic compensation (Fig. 1c). Interestingly, consistent with a previous report by Matta *et al*.^19^, we found that nearly complete loss-of-function of *dLrrk* (using three different *dLrrk* mutant combinations) had no significant consequence for any of the baseline electrophysiological properties at the NMJ (Supplementary Fig. 2a,b). These mutant combinations disrupt *dLrrk* transcription but do not affect transcription of neighbouring *GluRIID* or *GluRIIE* genes (Supplementary Fig. 2c–e). We also verified *GluRIIA* transcript levels and found no change (Supplementary Fig. 2e).

NMJs of *dLrrk* mutants have been reported to show a mild increase in the total number of synaptic boutons^20^; we observed a similar increase in the number of boutons in different allelic combinations of *dLrrk* mutant larvae (Supplementary Fig. 3a,b). We extended these analyses by assessing the number of synaptic release sites in *dLrrk* mutants both at the level of light microscopy and at the ultrastructural level. We found no differences in the total number of presynaptic release sites per NMJ between *dLrrk* mutant larvae and control larvae (Supplementary Fig. 3c–i). Similarly, heterozygous *dLrrk* mutant larvae showed no structural defects compared with control larvae (Supplementary Fig. 3i,j). These results indicate that the block of homeostatic response by loss of *dLrrk* is not likely to be a result of structural defects or defects in basal electrophysiological properties.

To determine the pre- and/or postsynaptic requirement for dLRRK, we turned to the UAS/Gal4 expression system in *Drosophila*^21^ to knock down dLRRK in a tissue-specific manner using transgenic RNA interference (RNAi) approach. Overexpression of a dLRRK-RNAi transgene (UAS-dLRRK-RNAi) in either muscle (using MHC-Gal4) or motoneurons (using BG380-Gal4) was capable of reducing the level of *dLrrk* transcript significantly and specifically in the corresponding tissues (Supplementary Fig. 4a,b). Our electrophysiological analysis indicated that overexpression of UAS-*dLrrk*-RNAi in muscles was sufficient to block the retrograde synaptic enhancement in *GluRIIA* mutants robustly, whereas overexpression of the same transgene in motoneurons had little effect (Fig. 1d-f). Moreover, we found that knockdown of dLRRK in postsynaptic muscles did not affect the baseline electrophysiological properties at the NMJ (Fig. 1e). Finally, structural analysis of larvae expressing UAS-*dLrrk*-RNAi postsynaptically did not show any change in the level or number of postsynaptic densities or presynaptic active zones per NMJ (Supplementary Fig. 3k).

The *Drosophila* larval NMJ expresses another form of synaptic homeostasis that can be triggered acutely on the scale of minutes^22^. When semi-dissected larvae are exposed to philantotoxin, GluRs can be readily blocked leading to a significant reduction of the size of miniature synaptic currents; however, over the course of 10–30 min, NMJs show a rapid and robust homeostatic compensation in presynaptic release^22^. Interestingly, this rapid development of homeostatic response appears to be independent of *de novo* protein synthesis^22^,^23^. We therefore predicted that this form of homeostatic plasticity might not be dependent on *dLrrk*. Our electrophysiological examination of *dLrrk* mutants supported this prediction. Following treatment of semi-intact larvae with philantotoxin, we found a robust expression of homeostatic plasticity in *dLrrk* mutants similar to what has been observed in wild-type larvae^22^ (Supplementary Fig. 5a,b).

In addition to highlighting the different requirements for the developmentally sustained versus the acutely induced homeostatic compensation, this finding rules out the possibility that *dLrrk* mutants might be fundamentally incapable of showing homeostatic compensation due to a secondary defect.

### LRRK2 can induce retrograde synaptic enhancement

**Figure 2:**
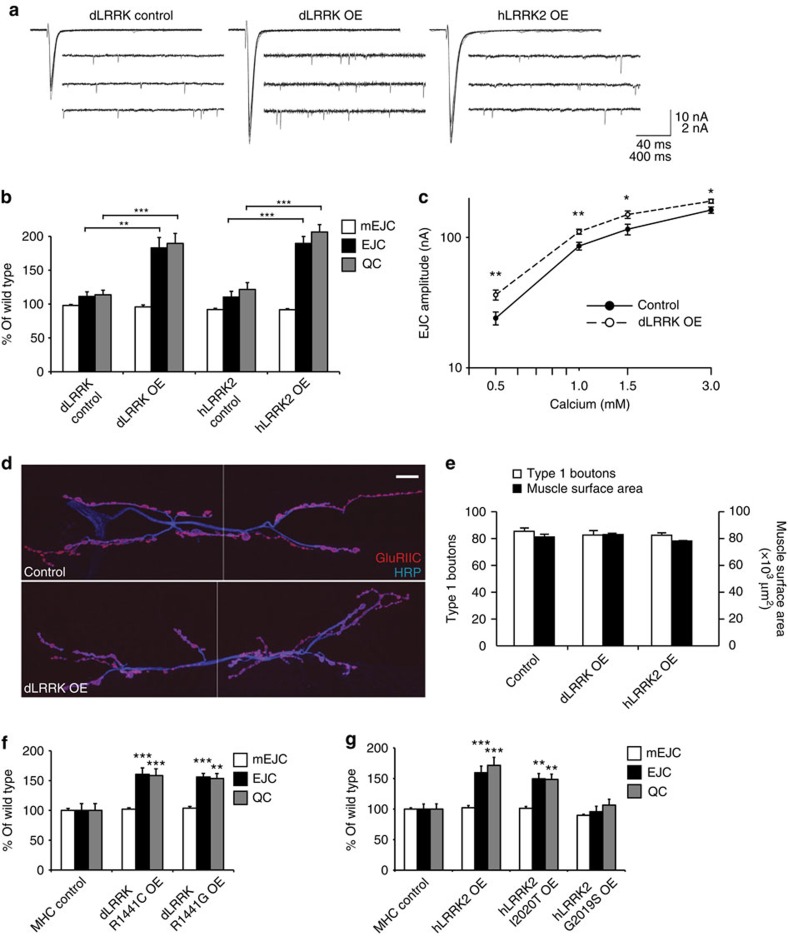
Postsynaptic overexpression of LRRK2 results in a retrograde enhancement in neurotransmitter release. (**a**) Representative traces of mEJCs and EJCs from dLRRK control (UAS*-dLRRK/+*), muscle overexpression of dLRRK (*G14-Gal4*/UAS*-dLRRK*) and muscle overexpression of hLRRK2 (*G14-Gal4*/*+*; +/UAS*-hLRRK2*). EJCs are ten superimposed consecutive traces recorded at 0.5 Hz; mEJCs represent continuous recordings. For details of the number of recordings and statistics, see Supplementary Table 1. (**b**) Quantification of mEJC, EJC and QC from the genotypes shown in **a** and control larvae for hLRRK2 overexpression (UAS*-hLRRK2*/+). *n*=21, 20, 20 and 28. (**c**) Quantification of EJCs at 0.5, 1.0, 1.5 and 3 mM external Ca^2+^ concentrations for control (*MHC-Gal4*/+), *n*=20, 10, 12 and 13, and dLRRK OE (UAS-*dLRRK/+; MHC-Gal4/+*), *n*=20, 11, 13 and 15 NMJs. (**d**) Muscle 6/7 NMJs double-stained with anti-GluRIIC (red) and anti-HRP (blue) in control (*MHC-Gal4*/+) and larvae overexpressing dLRRK in the muscle (+/UAS*-dLRRK*; *MHC-Gal4*/+). Scale bar, 10 μm. (**e**) Quantification of total number of type 1 boutons and muscle surface area from the genotypes shown in **d** and larvae overexpressing hLRRK2 in muscle (*MHC-Gal4*/UAS*-hLRRK2*). *n*=10, 10 and 10 NMJs. (**f**) Quantification of mEJC, EJC and QC from MHC control (*MHC-Gal4*/+), dLRRK^R1441C^ OE (UAS-dLRRK^R1441C^/+; *MHC-Gal4*/*+*) and dLRRK^R1441G^ OE (*MHC-Gal4*/UAS**-dLRRK^R1441G^). *n*=15, 18 and 18. See also Supplementary Table 1. (**g**) Quantification of mEJC, EJC and QC from MHC control (*MHC-Gal4*/*+*), hLRRK2 OE (*MHC-Gal4*/UAS-hLRRK2), hLRRK2^I2020T^ OE (*MHC-Gal4*/UAS**-hLRRK2^I2020T^) and hLRRK2^G2019S^ OE (*MHC-Gal4*/UAS**-hLRRK2^G2019S^). *n*=12, 13, 20 and 20. Error bars represent s.e.m. *P<0.05, **P<0.01 and ***P<0.001. See also Supplementary Table 1.

We have previously established that an increase in postsynaptic cap-dependent translation can lead to a retrograde enhancement in presynaptic release^4^. Thus, we asked whether genetic gain-of-function of dLRRK or the human transgene UAS-hLRRK would be sufficient to induce a retrograde enhancement in synaptic function in otherwise wild-type larvae. Indeed, postsynaptic overexpression of either transgene led to a large increase in EJCs without affecting the size of mEJCs, indicating a large increase in QC (Fig. 2a,b and Supplementary Fig. 5c). The robust increase in QC was seen using three different muscle drivers, MHC-Gal4, G14-Gal4 and 24B-Gal4, overexpressing either hLRRK2 or dLRRK (Supplementary Table 1). We repeated our recordings at different external calcium concentrations and found that EJCs where enhanced in response to overexpression of dLRRK over a range of calcium concentration from 0.5 to 3 mM (Fig. 2c and Supplementary Fig. 5d).

To understand the mechanism of action of LRRK2 further, we examined whether postsynaptic overexpression of LRRK2 influenced the number of synaptic boutons, presynaptic release sites, or the structure or density of postsynaptic GluR fields. We found no significant differences in these structural features at the NMJ between control larvae or those overexpressing dLRRK or hLRRK2 postsynaptically (Fig. 2d,e and Supplementary Fig. 6a–c). In addition, a detailed analysis of mEJC distributions revealed no changes associated with overexpression of dLRRK/hLRRK2 (Supplementary Fig. 6d,e). Consistently, we did not find any changes in GluRIIA levels at the NMJ when dLRRK was overexpressed in postsynaptic muscles (Supplementary Fig. 6f,g). These results indicated that the increase in synaptic strength associated with postsynaptic overexpression of dLRRK/hLRRK2 was most likely to be due to an increase in presynaptic neurotransmitter release probability or an enhancement in the size of readily releasable pool (RRP) of vesicles.

### Pathogenic mutations influence LRRK2's synaptic function

A number of LRRK2 pathogenic mutations have been linked to familial Parkinson's disease analysis; depending on the cellular context, these mutations have been reported to have wide ranging effects on LRRK2 function from gain-of-function mutations to dominant negative and loss-of-function^5^,^8^,^24^,^25^,^26^,^27^. To verify how pathogenic mutations in LRRK2 affect its ability to induce synaptic compensation, we tested four different transgenes: two kinase domain mutations (hLRRK2^G2019S^ and hLRRK2^I2020T^) and two Ras-of-complex–GTPase domain mutations (dLRRK^R1441G^ and dLRRK^R1441C^). Muscle overexpression of dLRRK^R1441G^ and dLRRK^R1441C^ induced synaptic compensation indistinguishable from that induced by wild-type dLRRK (Fig. 2f and Supplementary Fig. 6h), suggesting that the changes in GTPase activity associated with these mutations^28^,^29^ do not significantly interfere with the ability of dLRRK to induce synaptic compensation. However, overexpression of kinase mutant hLRRK2^G2019S^ failed to induce synaptic compensation, whereas overexpression of hLRRK2^I2020T^ transgene showed a trend towards reduced synaptic compensation compared with wild-type hLRRK2 (Fig. 2g and Supplementary Fig. 6i). We verified the expression level of the kinase mutant transgenes and found they express at a comparable level to wild-type hLRRK2 transgene (Supplementary Fig. 6j,k). As both transgenes are thought to influence the kinase function *in vitro*^25^,^26^,^30^, we set out to test whether kinase function is essential for LRRK2's ability to induce synaptic compensation. For this we overexpressed a kinase-dead transgene (dLRRK-3KD) that failed to induce any synaptic compensation, indicating a critical requirement for kinase function (QC for control, dLRRK overexpression and dLRRK-3KD overexpression was 35.56±2.65, 58.66±5.09 and 33.79±5.06, respectively; mean±s.e.m., *P*<0.01). As hLRRK2^G2019S^ has abnormally high kinase activity^10^,^19^,^25^,^31^, our findings suggest that, although kinase function is essential for LRRK2 to trigger synaptic compensation, abnormally high kinase function may also be detrimental for its ability to induce synaptic compensation. It is also possible that this mutation can affect other aspects of LRRK2 function or its ability to physically interact with other proteins, leading to disruption of LRRK2 synaptic function.

### dLRRK enhances the size of release ready pool of vesicles

**Figure 3:**
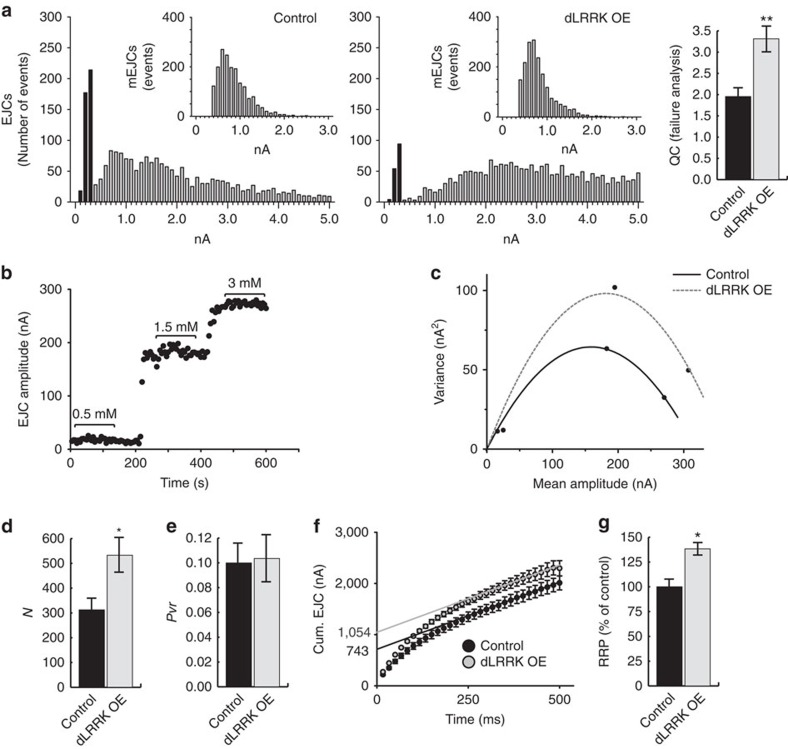
Postsynaptic overexpression of dLRRK increases pool of readily releasable vesicles. (**a**) Failure analysis of dLRRK OE larvae. Distribution of EJC amplitudes and mEJC amplitudes (inset) recorded under low external Ca^2+^ concentrations (0.25 mM) for control (*G14-Gal4*/+, *n*=2,572 EJCs and 1,784 mEJCs (left), and larvae overexpressing dLRRK in the muscle (*G14-Gal4*/UAS*-dLRRK*, *n*=3,024 EJCs and 1,828 mEJCs) (middle). Black bars indicate events that were considered as failures. (control *n*=409, dLRRK OE *n*=152). Quantification of QC (right). **P<0.001, Student's *t*-test. Error bars are s.e.m. (**b**) Representative (continuous recording) of EJC amplitudes in control (*MHC-Gal4/+*) larvae elicited at 0.2 Hz at the indicated Ca^2+^ concentrations. (**c**) Representative parabolic variance–mean plots for control (*MHC-Gal4*/*+*) (solid) and overexpression of dLRRK (UAS*-dLRRK*/*+*; *MHC-GAL4*/*+*) in muscle (dotted). (**d**,**e**) Quantal parameters for the number of ready-release vesicles (**d**) and the probability of vesicle release (**e**) for overexpression of dLRRK (UAS*-dLRRK*/+; *MHC-Gal4*/+) and control (*MHC-Gal4*/+). *n*=7 and 5. (**f**,**g**) Mean cumulative EJC plot with back extrapolation of linear fit from the last ten stimuli to time zero (**f**) and bar graph shows the relative size of RRP (**g**) for overexpression of control (*MHC-Gal4*/+) and dLRRK OE (UAS-*dLRRK/+; MHC-Gal4*/+). *n*=8 and 10. Recordings performed in 3 mM Ca^2+^ HL3 for 60 Hz, 30 stimuli. Error bars represent s.e.m. *P<0.05 and **P<0.01. See Supplementary Table 1.

To examine presynaptic release probability directly, we conducted failure analysis experiments. We found that postsynaptic overexpression of dLRRK led to a significant increase in QC, indicated by a smaller number of stimuli that failed to trigger EJCs at low extracellular calcium concentrations (Fig. 3a and Supplementary Fig. 7a,b). Failure analysis has revealed a similar increase in QC in GluRIIA mutant larvae as reported previously^3^. Mechanistically, presynaptic compensation in GluRIIA mutants is associated with an increase in the size of the RRP of vesicles^32^. As a similar synaptic compensation is triggered by postsynaptic overexpression of LRRK2, we conducted an assessment of the size of the RRP (*N*) and the probability of vesicle release (*P*_vr_) by recording EJCs over a range of external calcium concentrations from 0.5 to 3 mM (See methods). Parabolic fits to variance-mean plots estimated a significant increase in the size of RRP in larvae overexpressing dLRRK postsynaptic muscles (control 312.24±50.38 and overexpressed dLRRK 532.79±72.95; mean±s.e.m., *P*<0.05, *n*=8, 10), but revealed no significant difference in quantal size (q) or *P*_vr_ over this range of external calcium concentrations between the two genotypes (Fig. 3b–e). These results are consistent with the lack of an increase in *P*_vr_ associated with synaptic compensation in GluRIIA mutant larvae reported previously^32^. We also estimated the size of RRP by analysing cumulative high frequency-evoked synaptic currents, while larvae were bathed in 3 mM external calcium^33^. The results obtained in this experiment supported our analysis of variance-mean plots by indicating a significant increase in the amplitude of cumulative currents and a similar enhancement in the relative size of the RRP induced by postsynaptic overexpression of dLRRK (Fig. 3f,g).

Therefore, these results demonstrate that overexpression of dLRRK in postsynaptic muscles is sufficient to induce a retrograde synaptic enhancement at the NMJ and indicate that the primary mechanism underlying this enhancement is an increase in the size of RRP of vesicles.

### Cap-dependent translation is critical for dLRRK function

**Figure 4:**
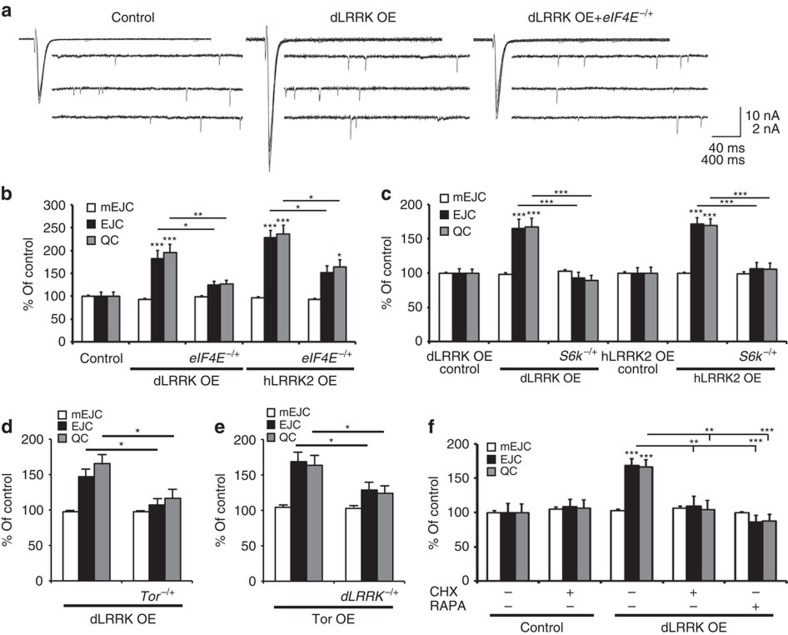
Retrograde synaptic enhancement by dLRRK and hLRRK2 is dependent on translation. (**a**) Representative traces of mEJCs and EJC from control (*G14-Gal4*/+), dLRRK muscle overexpression (*G14-Gal4*/UAS*-dLRRK*) and dLRRK muscle overexpression in *eIF4E* heterozygotes (*G14-Gal4*/UAS-*dLRRK*; *eIF4E*^*s058911*^/+). (**b**) Quantification of mEJC, EJC and QC from the genotypes shown in **a**, as well as hLRRK2 overexpression in the muscle (+/*G14-Gal4*; UAS*-hLRRK2*/+) and hLRRK2 muscle overexpression in *eIF4E* heterozygotes (+/*G14-Gal4*; UAS*-hLRRK2*/*eIF4E*^*s058911*^). *n*=13, 18, 16, 10 and 10. (**c**) Quantification of mEJC, EJC and QC from control larvae for dLRRK (UAS*-dLRRK/+*), larvae overexpressing dLRRK in muscle (*G14-Gal4*/UAS*-dLRRK*), dLRRK muscle overexpression in *S6k* heterozygotes (UAS*-dLRRK*/*G14-Gal4*; +/*S6k*^*l−1*^), control larvae for hLRRK2 (UAS-*hLRRK2*/+), hLRRK2 overexpression in muscle (*G14-Gal4*/+; +/UAS-*hLRRK2*) and hLRRK2 muscle overexpression in *S6k* heterozygotes (+/G14-Gal4; UAS-*hLRRK2*/ *S6k*^*1−1*^). *n*=21, 20, 20, 20, 28 and 20. (**d**) Quantification of mEJC, EJC and QC from larvae overexpressing dLRRK in muscle (*MHC-Gal4*/UAS*-dLRRK*) or in combination with *Tor* heterozygocity (UAS*-dLRRK*/*MHC-Gal4*; +/*Tor). n*=14 and 19. (**e**) Quantification of mEJC, EJC and QC from larvae overexpressing TOR in muscle (*MHC-Gal4*/UAS*-TOR*) or in combination with *dLrrk* heterozygocity (UAS*-TOR*/*MHC-Gal4*; +/*dLrrk*^*e03680*^*). n*=16 and 19. (**f**) Quantification of mEJC, EJC and QC from control larvae (*MHC-Gal4*/+) and larvae overexpressing dLRRK in muscle (+/UAS*-dLRRK*; *MHC-Gal4*/+) grown on normal food or food supplemented with 500 mg ml^−1^ cycloheximide or 1 μM rapamycin for 12 h. *n*=10, 10, 10, 8 and 8. Error bars represent s.e.m. *P<0.05, **P<0.01 and ***P<0.001. See also Supplementary Table 1.

Our results suggest that the function of LRRK2 in the retrograde regulation of synaptic function is most probably reliant on its ability to influence cap-dependent translation. A critical and rate limiting step in translation initiation is the binding of the eIF4E to the 5′-end cap structure of mRNAs^34^. Therefore, we tested whether LRRK2's action was sensitive to levels of eIF4E. We found that heterozygosity for eIF4E, which has no effect on baseline synaptic activity^4^, resulted in a reduced ability of dLRRK or hLRRK2 to induce a retrograde enhancement in synaptic strength (Fig. 4a,b). Moreover, as we have shown previously, postsynaptic S6K (p70 S6 ribosomal protein kinase) activity is also essential for normal homeostatic synaptic compensation in GluRIIA mutants^4^; therefore, we tested whether LRRK2-induced synaptic enhancement is also critically dependent on the availability of S6K and found that removal of one gene copy of S6K was sufficient to block the ability of either dLRRK or hLRRK2 to induce synaptic enhancement at the NMJ (Fig. 4c). We extended these genetic interactions further by showing that postsynaptic dLRRK gain-of-function at the synapse was also dominantly suppressed by the removal of one gene copy of *Tor* (Fig. 4d). Finally, we tested whether TOR gain-of-function would be affected by loss of *dLrrk*. As we have shown previously, muscle overexpression of TOR can lead to a significant enhancement of synaptic release at the NMJ (Fig. 4e and Penney *et al*.^4^). Heterozygosity of *dLrrk* was sufficient to significantly block this increase (Fig. 4e), suggesting that TOR and dLRRK functionally interact. These data also support the notion that endogenous dLRRK function is required for normal postsynaptic translational pathways to function effectively.

In further support for the dependence of the synaptic action of dLRRK on translation, we found that feeding third-instar larvae pharmacological blockers of translation, cycloheximide or rapamycin for 12 h (before recording) was sufficient to suppress the enhancement in QC caused by postsynaptic overexpression of dLRRK, whereas the same treatment did not affect the base line electrophysiological properties at the NMJ (Fig. 4f). These results demonstrate that the retrograde enhancement of synaptic strength induced by LRRK2 requires *de novo* protein synthesis and is heavily dependent on cap-dependent translation. In addition, these results indicate that maintenance of retrograde synaptic enhancement induced by LRRK2 requires sustained protein synthesis, as does homeostatic synaptic compensation in *GluRIIA* mutant larvae^4^.

### Furin 1 is a translational target of dLRRK

**Figure 5:**
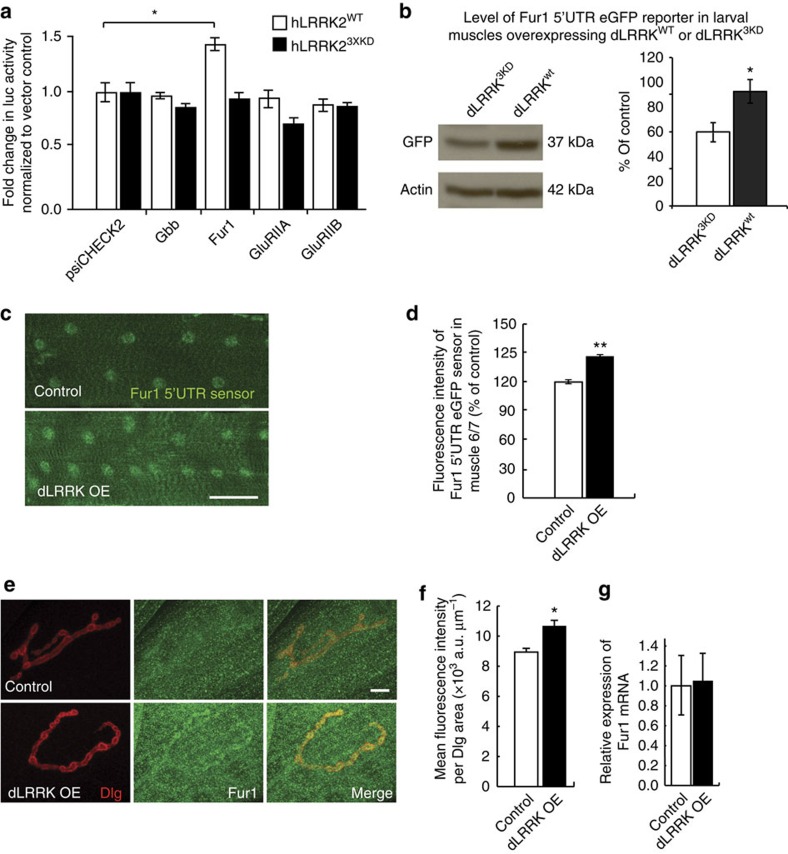
dLRRK and hLRRK2 promote cap-dependent translation in postsynaptic muscles. (**a**) Quantification of 5′-UTR luciferase reporter activity in response to co-transfection with either wild-type hLRRK2^wt^ or kinase-dead hLRRK2^3XKD^ normalized to psiCHECK2 response in HEK293T. *n*=3 experiments, *P=0.043. See also Supplementary Table 2. (**b**) Western blot analysis of *in vivo* Fur1-5′-UTR-eGFP reporter expression when co-expressed with either dLRRK^3KD^ (+/UAS-*Fur1*-5′-UTR-*eGFP*; UAS*-dLRRK*^*3KD*^/*MHC-Gal4*) or wild-type dLRRK^wt^ (UAS*-dLRRK*^*wt*^/UAS-*Fur1*-5′-UTR-*eGFP*; +/*MHC-Gal4*). Left: Western blotting probed with (top) anti-GFP and (bottom) anti-Actin as loading control. Right: quantification of Fur1-5′-UTR-eGFP expression normalized to actin levels. *n*=3 for each. *P<0.05. (**c**) Muscle 6 expression of Fur1-5′-UTR-eGFP in control (UAS-*Fur1*-5′-UTR-*eGFP*/*24B-GAL4*) and dLRRK-overexpressing larva (UAS*-dLRRK*/+; *24B-GAL4*/UAS-*Fur1*- 5′-UTR-*eGFP*). Scale bar, 100 μm. (**d**) Quantification of fluorescence intensity of muscles 6 and 7 normalized to muscle volume and expressed as a percentage of control. *n*=20 and 15 muscles, **P=0.001. (**e**) Muscle 4 NMJs stained with anti-Discs large (Dlg, red), anti-Fur1 (green) in control (*MHC-Gal4*/+) and dLRRK muscle overexpression larvae (+/UAS*-dLRRK*; *MHC-Gal4*/*+*). Scale bar, 10 μm. (**f**) Quantification of fluorescence intensity within Dlg area of genotypes in **e**. *n*=18 and 16 NMJs. *P=0.044. (**g**) Quantification of *Fur1* mRNA expression by qPCR. *n*=3 technical replicates. Error bars represent s.e.m. Student's *t*-test.

What are the potential translational targets of dLRRK in postsynaptic muscles? To address this question we set out to use a combination of *in vitro* and *in vivo* translation assays looking for potential translational targets that may be regulated by LRRK2. Complexity of the secondary structure of 5′-UTR renders mRNAs more dependent on the function of the cap-binding protein complex and, in particular, the ability of eIF4A to unwind double-stranded RNA, which facilitates the bindings of the ribosome to the mRNA during translation initiation^35^. Therefore, we first conducted a luciferase-based *in vitro* translation reporter assay testing over thirty-five 5′-UTRs belonging to *Drosophila* mRNAs with complex secondary structure and/or relevance to synaptic structure and function (Supplementary Table 2). As expected, the more complex 5′-UTRs (as measured by the free energy (ΔG) of RNA secondary structure calculated with UNAfold software^36^) generally suppressed luciferase activity in HEK293T cells compared with less complex 5′-UTRs (Supplementary Table 2), confirming that complexity of 5′-UTR negatively correlates with translation. Interestingly, only a few of 5′-UTRs exhibited significant response to LRRK2 (Supplementary Table 2), with the 5′-UTR belonging to the proprotein convertase Furin 1 (Fur1) showing the strongest response (Fig. 5a). We verified that indeed this induction in translation was kinase dependent, as a kinase-dead form of hLRRK2 did not increase Fur1 reporter activity (Fig. 5a).

We next used a transgenic green fluorescent protein (GFP) reporter containing the Fur1 5′-UTR (UAS-Fur1-5′-UTR-eGFP) that was previously shown to be sensitive to TOR activity in postsynaptic muscles^4^. Similarly, we found that overexpression of dLRRK in postsynaptic muscles led to an ∼25% increase in reporter activity, suggesting that gain-of-function of dLRRK can induce cap-dependent translation in larval muscles (Fig. 5b–d and Supplementary Fig. 8a). Finally, to verify that dLRRK can regulate endogenous Fur1 protein levels, we conducted immunohistochemistry at the NMJ using a previously characterized antibody against Fur1 (ref. 37). Overexpression of dLRRK led to a significant increase in Fur1 levels in postsynaptic muscles and enhanced Fur1 accumulation at postsynaptic sites based on fluorescence intensity measurements (Fig. 5e–g). We also verified, using quantitative PCR (qPCR), that this effect was not accompanied by a change in Fur1 mRNA levels (Fig. 5g).

**Figure 6:**
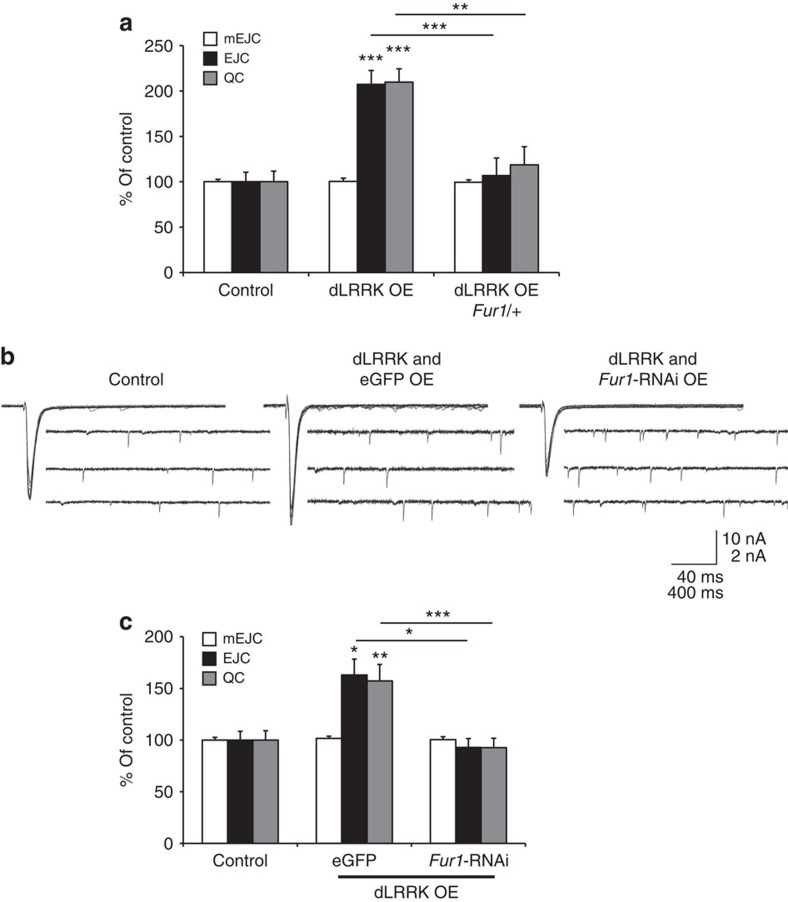
Postsynaptic Fur1 is required for enhancement of synaptic transmission by dLRRK. (**a**) Quantification of mEJC, EJC and QC from control (*MHC-Gal4*/*+*), dLRRK OE (UAS*-dLRRK*/*+*; *MHC-Gal4*/*+*) and dLRRK OE *Fur1*/+ (UAS*-dLRRK*/*+*; *MHC-Gal4*/*Fur1*^*rl205*^). *n*=10, 10 and 10. (**b**) Representative traces for mEJC and EJC in control (*MHC-Gal4/*+), dLRRK and eGFP co-expression in muscle (*+*/UAS*-dLRRK*; *MHC-Gal4*/UAS*-eGFP*) and dLRRK muscle overexpression with *Fur1* RNAi (+/UAS*-dLRRK*; *MHC-Gal4*/UAS*-Fur1-RNAi*). (**c**) Quantification of mEJC, EJC and QC from the genotypes shown in **a**. *n*=18, 18 and 18. Error bars represent s.e.m. *P<0.05, **P<0.01 and ***P<0.001. See also Supplementary Table 1.

Next, we tested the relevance of Fur1 to the ability of postsynaptic dLRRK to induce synaptic enhancement in two ways. First, we tested whether removal of one gene copy of *Fur1* gene can influence the action of dLRRK. For this we used an allele of *Fur1*, where a transposon insertion has disrupted the normal transcription of *Fur1* gene (Supplementary Fig. 8b). We found in larvae heterozygous for *Fur1* the retrograde synaptic enhancement normally induced by postsynaptic overexpression of dLRRK was significantly reduced (Fig. 6a). Next, we set out to test the consequence of postsynaptic knockdown of Fur1 using transgenic RNAi approach. We first examined the effectiveness of a Fur1-RNAi transgene (Supplementary Fig. 8c). Next, we tested the consequence of muscle overexpression of UAS-Fur1-RNAi on dLRRK gain-of-function. We found that co-expression of UAS-Fur1-RNAi together with UAS-dLRRK strongly inhibited the retrograde synaptic enhancement that is normally caused by postsynaptic overexpression of UAS-dLRRK, whereas co-expression of UAS-eGFP (as a control transgene) did not have any adverse effects (Fig. 6b,c). Thus, these results support a model whereby dLRRK exerts its function in the regulation of retrograde synaptic enhancement at the NMJ at least in part by promoting postsynaptic translation of Fur1.

### Postsynaptic LRRK2^G2019S^ blocks synaptic compensation

**Figure 7:**
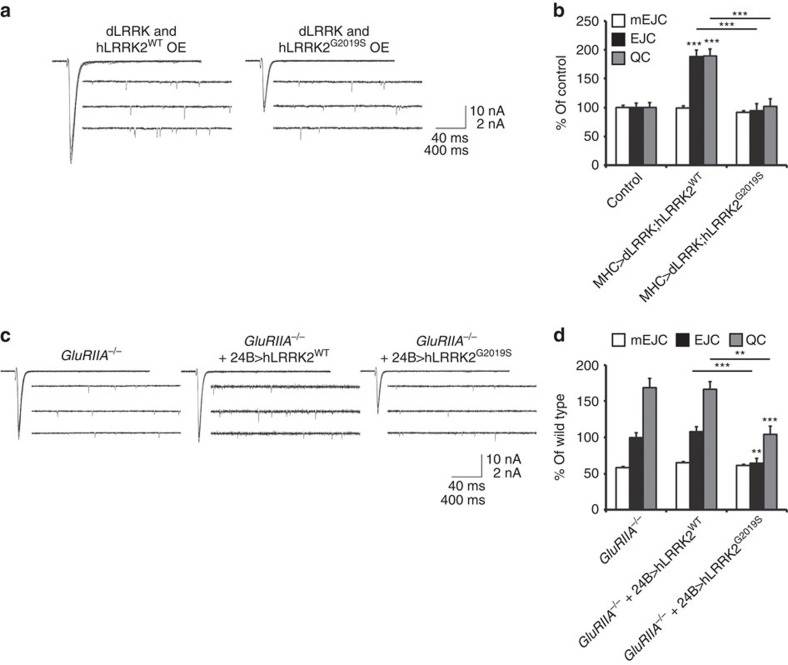
hLRRK2^G2019S^ inhibits retrograde signalling. (**a**) Representative traces of mEJCs and EJC from larvae co-expressing dLRRK with hLRRK2^wt^ (UAS-dLRRK/+; MHC-GAL4/UAS-hLRRK2^wt^) and larvae co-expressing dLRRK with hLRRK2^G2019S^ (UAS-dLRRK/+; MHC-GAL4/UAS-hLRRK2^G2019S^). (**b**) Quantification of mEJC, EJC and QC from genotypes in **a** as well as control (MHC-Gal4/+). *n*=20, 20 and 22. (**c**) Representative traces of mEJCs and EJC from *GluRIIA* mutant larvae (*Df(2L)cl-h4/GluRIIA*^*SP16*^) or *GluRIIA* mutant larvae expressing either wild-type or mutant hLRRK2 in muscle (*Df(2L)cl-h4/GluRIIA*^*SP16*^; 24B-Gal4*/*UAS-hLRRK2^wt^ and *Df(2L)cl-h4/GluRIIA*^*SP16*^; 24B-Gal4*/*UAS-hLRRK2^G2019S^). (**d**) Quantification of mEJC, EJC and QC from **c**. *n*=20, 20 and 22. Error bars represent s.e.m. **P<0.01 and ***P<0.001.

The failure of hLRRK2^G2019S^ in inducing a retrograde enhancement in neurotransmitter release at the NMJ prompted us to test the idea that this mutant transgene might in fact interfere with the normal function of endogenous dLRRK and act as dominant negative. We tested this idea in two ways. First, we examined whether the mutant transgene could interfere with the function of the wild-type transgene when both were expressed simultaneously. Indeed, we found that coexpression of mutant hLRRK2^G2019S^ led to a strong suppression of dLRRK's ability to induce a typical enhancement in presynaptic release (Fig. 7a,b). Second, we tested whether overexpression of hLRRK2^G2019S^ could interfere with the ability of the NMJ to undergo retrograde synaptic compensation in *GluRIIA* mutants. Again, in support of our hypothesis, we found that postsynaptic overexpression of hLRRK2^G2019S^ in *GluRIIA* mutant larvae led to a significant suppression of the retrograde synaptic compensation that is normally observed in these mutants (Fig. 7c,d), suggesting that hLRRK2^G2019S^ can interfere with the function of endogenous dLRRK.

## Discussion

Our findings demonstrate that genetic removal of *dLrrk* blocks the retrograde homeostatic synaptic compensation that is normally operating at the *Drosophila* larval NMJ. Normal activity of dLRRK is required in postsynaptic muscles, rather than in presynaptic neurons, to ensure normal homeostatic response. In addition, we find that postsynaptic overexpression of dLRRK is sufficient to induce a retrograde enhancement in synaptic release. Therefore, these results establish dLRRK as a regulator of synaptic strength through its ability to regulate homeostatic compensation and to induce retrograde synaptic enhancement. Interestingly, when the human LRRK2 is transgenically expressed in postsynaptic muscles, it also triggers a retrograde enhancement in presynaptic neurotransmitter release that is indistinguishable from the effect of *Drosophila* transgene. Based on these results, we propose that changes in LRRK2 function in neurons may lead to the disruption of homeostatic compensation and/or dysregulation of synaptic strength and ultimately undermine normal synaptic activity in neural circuits.

A recent report has provided evidence for interaction between LRRK2 and the ribosomal protein S15, suggesting that LRRK2 directly phosphorylates the ribosomal protein S15 (ref. 10). We did not establish or rule out a potential interaction between dLRRK and the ribosomal protein S15 at the NMJ; however, our results indicate that LRRK2 can effectively promote cap-dependent translation *in vitro* and *in vivo*. Strong genetic interaction with initiation factor eIF4E indicates that promotion of the translation by LRRK2 is at least in part dependent on its ability to promote translation at the level of initiation, a function that is distinct from LRRK2's ability to enhance ribosomal protein function. We have postulated that the specificity of LRRK2's function may lie in the translational regulation of a specific set of mRNAs that contain highly complex 5′-UTRs, as such mRNAs are expected to rely more heavily on the availability of the cap-binding protein complex^35^. In support of this idea, we have demonstrated that Fur1 mRNA, with a highly complex 5′-UTR, is translationally regulated by LRRK2 in postsynaptic muscles. In addition, we demonstrate that genetic knockdown of Fur1 in postsynaptic muscles is sufficient to block the ability of LRRK2 to induce a retrograde enhancement in synaptic release, validating our approach. Further experiments are needed to determine translational targets of hLRRK2 in the mammalian nervous system, which may lead to new therapeutic approaches through identification of novel drug targets for Parkinson's disease.

The presence of many putative functional motifs in LRRK2 protein predicts multiple functions for this large intracellular protein. As such, LRRK2 has been implicated in a number of processes from regulation of mitochondrial function and autophagy to the regulation of endocytic pathways and synaptic growth^19^,^20^,^38^,^39^,^40^,^41^. The *Drosophila* larval NMJ has been used previously as a model to study the synaptic role of LRRK2. In an elegant biochemical and electrophysiological study, Matta *et al*.^19^ demonstrated that, under conditions of high-frequency stimulation or sustained depolarization (high K^+^ treatment), dLRRK plays a role in the regulation of endocytosis in presynaptic motoneurons; LRRK2 exerts this action by phosphorylating Endophilin, a membrane-associated protein that is required for endocytosis of synaptic vesicles. Under these conditions, too much phosphorylation or too little phosphorylation of Endophilin by dLRRK appears to disrupt the normal process of endocytosis. Nevertheless, under resting physiological conditions, *dLrrk* mutants and wild-type larvae were reported to show similar evoked synaptic activity^19^. This is consistent with our findings that baseline electrophysiological properties and the number of synaptic release sites are not affected in *dLrrk* mutant larvae.

In contrast to what Matta *et al*.^19^ and our findings suggest, Lee *et al*.^20^ have reported a reduction in baseline electrophysiological properties, in particular a reduction in QC, in *dLrrk* mutants. In addition, Lee *et al*.^20^ did not report an increase in QC associated with overexpression of LRRK2. These inconsistencies may be partially due to differences in recording conditions^20^. Indeed, we find that at 3 mM calcium concentration, the increase in QC in response to postsynaptic overexpression of dLRRK becomes less exaggerated and statistically insignificant. Synaptic compensation in GluRIIA mutant larvae shows a similar decline with increasing levels of extracellular calcium^32^; although GluRIIA larvae show accurate matching of wild-type EJCs up to 1.5 mM external calcium, they fail to produce EJCs as large as wild-type EJCs at higher external calcium concentrations^32^. A simple interpretation of this phenomenon is that the closer the synapse is to its maximal probability of release, the harder it becomes to enhance the release further with increasing external calcium. Release probability is a complex and tightly regulated property of synapses that depends primarily on calcium influx and availability of releasable vesicles^42^. Studies of short-term facilitation both at the NMJ and at central synapses have produced a wealth of data that sheds light into mechanisms influencing release probability^43^. At synapses with low initial probability of release, if the presynaptic axon is stimulated twice within a short interval, the second stimulus evokes a larger synaptic response, referred to as paired pulse facilitation (PPF)^43^. Many synapses that show strong PPF at low calcium concentrations fail to show PPF at high external calcium concentrations^44^. Consistently, we find that over a range of external calcium concentrations from 0.5 to 1.0 mM, LRRK2 overexpression gives rise to a nearly linear and large increase in QC; however, as the external calcium is increased, while the increase in evoked synaptic currents persists, the degree of this increase deviates from linearity. Finally, we examined the effect of LRRK overexpression by conducting fluctuation analysis over a wide range of calcium concentrations; fitting variance–mean curves corresponding to varying calcium concentrations revealed a significant increase in the size of RRP of vesicles when LRRK was overexpressed in postsynaptic muscles, a change that appears qualitatively similar to what has been reported in *GluRIIA* mutant larvae^32^. Therefore, we propose that postsynaptic LRRK2 activity influences release probability by regulating RRP of vesicles at presynaptic terminals.

Although it is difficult to fully recapitulate the effect of pathogenic mutations on the normal function of LRRK2 in patients, our findings suggest that changes in GTPase activity of LRRK2 may be less critical for its synaptic function. However, we find that both loss of kinase activity and abnormally increased kinase activity in hLRRK^G2019S^ might hinder LRRK2's ability to induce synaptic compensation. We speculate, based on the model proposed by Matta *et al*. (2012), that increased kinase activity in response to strong overexpression of LRRK2 may lead to hyperphosphorylation and inhibition of a postsynaptic target protein; this in turn could negatively influence retrograde signalling at the NMJ. Indeed, muscle overexpression of LRRK2^G2019S^ interferes with wild-type function of dLRRK at the NMJ and blocks retrograde synaptic compensation in GluRIIA mutants. Accumulating evidence points to defects in synaptic transmission and alteration of synaptic strength as some of the earliest manifestations of disease in the case of neurodegenerative diseases such as Alzheimer's, Parkinson's and Huntington's disease^45^,^46^,^47^,^48^. Interestingly, our analysis of pathogenic mutations in LRRK2 bring to forefront a strong link between *Lrrk2* mutations and synaptic dysfunction, and suggests that both gain-of-function or loss-of-function mutations may lead to dysregulation of synaptic homeostasis and destabilize circuit function.

## Methods

### Fly stocks

Flies were cultured on standard medium at 25 ^o^C. *w*^*1118*^ (wild type), *dLrrk*^EX1^ (ref. 49), *eIF4E*^*s058911*^ (ref. 50), UAS-Fur1-RNAi (Stock# 25837) and *Fur1*^*rl205*^ were obtained from the Bloomington *Drosophila* Stock Center. *dLrrk*^*e03680*^ was obtained from the Harvard Medical School Stock Collection. UAS-dLRRK, UAS-hLRRK2^G2019S^ and UAS-dLRRK^3KD^ (ref. 9) were gifts from B. Lu. UAS-hLRRK2^I2020T^, UAS-dLRRK^R1441C^, UAS-dLRRK^R1441G^ and UAS-hLRRK2^wt^ (ref. 51) were gifts from D. Park. *GluRIIA*^*SP16*^, UAS-GluRIIA^M/R^ (ref. 18) and *Df(2l)cl-h4* (ref. 3) were gifts from C. Goodman. UAS-TOR, *Tor*^*E161K*^ (ref. 52) and *Tor*^*ΔP*^ (ref. 53) were gifts from T.P. Neufeld. *S6k*^*l*-*1*^ (ref. 54) was a gift from N.S. Moon. Motor neuron Gal4 drivers were BG380-Gal4 (ref. 55), OK371-Gal4 (ref. 56) and OK6-Gal4 (ref. 57). Muscle Gal4 drivers were MHC-Gal4 (ref. 58), G14-Gal4 (ref. 57) and 24B-Gal4 (ref. 21). UAS-dLRRK-RNAi was obtained from the Vienna *Drosophila* Research Center. UAS-eGFP and UAS-Fur1-5′-UTR-eGFP were described previously^4^.

### Immunostaining

Wandering third-instar larvae were dissected, internal organs removed and stretched with insect pins in ice-cold 0-mM Ca^2+^ Haemolymph-like saline (HL3) as previously described^58^. Larvae were fixed for 10 min with ice-cold 4% paraformaldehyde in PBS solution^59^ or 3 min in ice-cold methanol for GluRIIA staining, incubated overnight with primary antibodies at 4 °C and incubated with fluorescence-conjugated secondary antibodies at room temperature for 2 h. Primary antibodies were used in the following concentrations: mouse anti-Dlg 1:500 (ref. 55), rabbit anti-Synaptotagmin 1:2,000 (ref. 60), rabbit anti-DGluRIIC 1:2,000 (ref. 16), mouse anti-Brp (Nc82) 1:250, mouse anti-GluRIIA 1:250 (Developmental Studies Hybridoma Bank, USA), rabbit anti-Fur1 1:500 (ref. 37) and Alexa Fluor 647-conjugated goat anti-horseradish peroxidase (HRP) 1:250 (Jackson ImmunoResearch Laboratories Inc.). Secondary antibodies were used in the following concentrations: Alexa Fluor 488-conjugated goat anti-mouse 1:500, Alexa Fluor 488-conjugated goat anti-rabbit 1:500, Cy3-conjugated goat anti-mouse 1:500 and Cy3-conjugated goat anti-rabbit 1:500 (Amersham Bioscience).

### Electrophysiology

Wandering third-instar larvae were dissected in cold HL3 solution without Ca^2+^ following standard protocol^58^. The spontaneous (mEJC) and evoked (EJC) membrane currents were recorded from muscle 6 in abdominal segment A3 with standard two-electrode voltage-clamp technique^61^. All recordings were performed at room temperature in HL3 solution containing 0.5 mM Ca^2+^ unless otherwise indicated. The current recordings were collected with AxoClamp2B amplifier (Molecular Devices Inc.) using Clampex 9.2 software (Molecular Devices Inc.). Nerve stimulation was delivered through a suction electrode, which held the cut nerve terminal cord. In all voltage clamp recordings, muscles were held at −80 mV. The holding current was <5 nA for 90% of the recordings and we rejected any recording that required >10 nA current to maintain the holding potential. All records were subjected to 1 kHz low-pass filtering during acquisition. The amplitudes of mEJC and EJC were measured using Mini Analysis 6.0.3 software (Synaptosoft) and verified by the eye. QC was calculated by dividing the mean EJC amplitude by mean mEJC amplitude. The recording traces were generated with Origin 7.5 software (Origin Lab).

For the rapamycin or cycloheximide treatment, larvae were moved to food plates supplemented with 1 μM rapamycin or 500 mg ml^−1^ cycloheximide (Fisher Scientific). Third-instar larvae were grown for at least 12 h in drug containing food before being harvested for electrophysiological analysis as above.

### Failure analysis

In failure analysis the postsynaptic current is measured in response to repeated presynaptic stimulation at low calcium concentrations. Under these conditions, the proportion of stimuli that fail to trigger a postsynaptic response is negatively correlated with presynaptic release probability^62^. The recordings for failure analysis were done in 0.25 mM Ca^2+^ HL3 solution. The stimulation was applied repeatedly for more than 300 times (1 Hz). We considered an evoked response a failure if an event were within the distribution of baseline noise measurements and distinct from the distribution of mEJC amplitudes. For each NMJ, baseline noise was measured by measuring the amplitude difference in a 30-ms window in the prestimulus interval of at least eight events. The number of failure of evoked currents (*N*_0_) and total number of evoked events (*N*) were used to calculate QC according to the following formula: QC=ln (*N*/*N*_0_).

### Measurement of the size of the RRP

The size of the RRP was measured using a previously described method of cumulative EJC amplitudes^33^ that has been applied at the *Drosophila* larval NMJ^63^. Muscle 6 was voltage clamped at −80 mV and EJC amplitudes were measured in 3 mM Ca^2+^ HL3 solution (60 Hz, 30 stimuli). The cumulative EJC amplitude at time zero was measured by back-extrapolating a line fit to the last ten stimuli of each stimulus train to time zero. The number of release-ready vesicles was calculated by dividing the cumulative EJC amplitude at time zero by the mean mEJC amplitude of each NMJ.

### Analysis of variance–mean plots

Current amplitudes were measured at membrane potentials clamped at −80 mV with perfusion of different calcium concentrations of HL3 from 0.5 to 3 mM (⩾20 EJCs each, 0.2 Hz). The mean EJC amplitude is determined by *I*=*NP*_*vr*_*q* where *I* is the mean EJC amplitude, *N* is the number of ready-release vesicles, *P*_*vr*_ is the vesicular release probability and *q* is the quantal size. EJC variance was calculated by Var (*I*)=(1/(*n*−1)) ∑(*I*_*i*_−*I*_mean_)^2^ and variance–mean plots were fitted for each NMJ to the parabolic function Var (*I*)=*I*^2^/N+*qI* (ref. 64). The values *P*_*vr*_ and *q* were calculated by the equations: *P*_*vr*=_*I* (*B*/*A*) (1+CV^2^) and *q*=*A*/(1+CV^2^). CV^2^ is the coefficient of variation of EJC amplitudes at a specific calcium concentration calculated as CV^2^=(EJC s.d./EJC mean amplitude)^2^, and *A* and *B* were determined by parabolic fits^65^.

### Western blot analysis

Muscle tissue (without the nervous system and motor axons or imaginal discs) was isolated from wandering third-instar larvae dissected in cold HL3. Western blot analysis was performed according to manufacturer's protocols using the following primary antibodies: rabbit anti-phospho-4E-BP (T37/46) and rabbit anti-nonphospho-4E-BP (T46), both 1:1,000 (Cell Signaling), rabbit anti-hLRRK2 (3515-1) 1:1,000 (Epitomics), rabbit anti-GFP 1:1,000 (Molecular Probes) and mouse anti-Actin 1:2,000 (Chemicon). Protein bands were visualized using SuperSignal West Pico Chemiluminescent Substrate (Thermo Scientific). The gel images were scanned and band intensities were quantified using MetaMorph software (Molecular Devices). The following antibodies were used for hLRRK2 overexpression and hLRRK2 short hairpin RNA (shRNA) western blottings: anti-eIF4G (2498) from Cell Signaling, mouse monoclonal anti-eIF4A was a gift from Hans Trachsel (Bern, Switzerland)^66^, eIF4E: mouse anti-eIF4E (610270) from BD Biosciences, anti-4E-BP1 (53H11) Rabbit monoclonal antibody (9644) from Cell Signaling, anti-Phospho 4E-BP1 (236B4) Rabbit monoclonal antibody (2855) from Cell Signaling and monoclonal anti-β-actin (A5441) from Sigma.

### Imaging and data analysis

For synaptic bouton quantifications, muscle 4 and muscle 6/7 from segment A3 were analysed as previously described^58^, with the following modifications: NMJs were co-stained with anti-Dlg, anti-Syt and anti-HRP antibodies, and the counts were performed with a × 63/1.4 oil-immersion objective using an epi-fluorescence microscope Zeiss Imager Z1 (Carl Zeiss, Inc.). Synapses were imaged using a Confocor LSM 510 META on an Axiovert 200M inverted microscope and a LSM710 Confocal (Carl Zeiss, Inc.). Settings were optimized for detection without saturation of the signal. Images were obtained at room temperature using a Zeiss Plan-Apochromat × 63/1.4 oil-immersion objective (Carl Zeiss, Inc.). Images were cropped for figures using Photoshop CS5 (Adobe Inc.). For fluorescence quantifications, *z*-stacks were obtained from segment A3 NMJs using identical settings within each experiment. The fluorescence signal per synaptic area for each marker in maximum projections of the *z*-stacks was determined using MetaMorph software (Molecular Devices). HRP signal was used to delineate synaptic area for GluRIIA and GluRIIC quantifications, whereas Dlg area was used to quantify Fur1 fluorescence. Active zones were quantified by counting Brp puncta in maximum projections from confocal *z*-stacks. Fluorescence signal per muscle volume was determined using Imaris software (Bitplane).

### Calculation of ΔG for 5′-UTR

5′-UTR sequence release 5.29 was obtained from www.flybase.org^67^ and input into the Unified Nucleic Acid Folding and hybridization package (UNAfold)^36^ package to obtain free energy (ΔG) values. Calculations were performed at a physiologically relevant temperature of 25 °C.

### 5′-UTR luciferase reporter assay

The Fur1 and Gbb luciferase reporters were described previously^4^. The 5′-UTRs of all luciferase reporters were PCR amplified from complementary DNA obtained from *w*^*1118*^ larvae and cloned into the NheI site of psiCHECK-2 vector (Promega) upstream of the *Renilla* luciferase gene. HEK293T cells were transfected with control or 5′-UTR Luciferase sensors with or without co-transfection with hLRRK2 plasmid (hLRRK-WT (Addgene#17609)^25^ was a gift from Ted Dawson and hLRRK2-3xKD (Addgene#25366)^68^ was a gift from Mark Cookson) using Lipofectamine 2,000 (Invitrogen). After 48 h, cells were harvested and the luciferase activity was measured with the Dual Luciferase Reporter Assay System (Promega) on a Lumat Single Tube Luminometer LB 9507 (Berthold Technologies). *Renilla* luciferase activity was normalized to firefly luciferase for each transfection to control for transfection and expression levels. Luciferase reporter response in hLRRK co-transfected wells were compared with Luciferase reporter alone, to obtain the fold response to hLRRK2-WT or hLRRK2-3xKD.

### CAP and IRES luciferase reporter assay

hLRRK2-WT (Addgene: 17609) was transfected into cells. Two days after transfection, cells were transfected with firefly luciferase reporter mRNAs encoding control, EMCV IRES or HCV IRES at the 5′-UTR together with *Renilla* luciferase mRNA as a transfection control and lysed 18 h post transfection. Luciferase activities were determined using Dual-Luciferase Reporter Assay (Promega).

### Quantitative PCR

RNA was extracted from five larvae (third instar) with the Qiagen RNA Easy Plus kit (Cat No. 74134). cDNA was produced using the BioRad iScript cDNA synthesis kit (Cat No. 170-8891). qPCR data were generated using the BioRad Ssofast Evagreen Supermix (Cat. No. 175-5211) and the Illumina Eco qPCR system. qPCR primers used were as follows:

*Fur1* forward: 5′- AGGAATATGCAGCAGGTGGG -3′, *Fur1* reverse: 5′- TGCACTCTAAGCACTTGCGA -3′; tubulin control forward: 5′- TGTCGCGTGTGAAACACTTC -3′, tubulin control reverse: AGCAGGCGTTTCCAATCTG -3′; *dLrrk*^*e03680*^ forward: 5′- AGATCAACCCCTTTGCTCCT -3′, *dLrrk*^*e03680*^ reverse: 5′- AGCTTAACCGTGCTTCCTGA -3′; *dLrrk*^*ex1*^ mutation forward: 5′- AGACAATGTTCCGCTGATCG -3′, *dLrrk*^*ex1*^ mutation reverse: 5′- CAGAGCTCTTGGTGGATGACT -3′; *GluRIIA* forward: 5′- TTCAATCCCTCGGCCTTCAC -3′, *GluRIIA* reverse: 5′- GTCCGGTAATCAGAGCCCAG -3′; *GluRIID* forward: 5′- TACTCGAATACCAGAGGACGGA -3′, *GluRIID* reverse: 5′- TGATGAGGCCCAGGCGAATG -3′; *GluRIIE* forward: 5′- CCATAGGTCTGCTCACCGAC -3′, *GluRIIE* reverse: 5′- CAGCGATGCCAGTCTCTAGC -3′

### Metabolic radiolabelling

Cells were incubated in methionine/cysteine-free DMEM (GIBCO 21013) containing 10% dialysed fetal bovine serum (GIBCO 26400) for 30 min. Cell media was replaced with the medium containing 10 μCi/ml [^35^S]methionine/cysteine for 30 min. Cells were lysed in Laemmli buffer. [^35^S]Methionine/cysteine incorporation was determined by trichloroacetic acid precipitation followed by scintillation counting.

### shRNA silencing

Lentiviral vectors expressing shRNAs were purchased from Sigma. shRNA vectors encoding shRNAs targeting human LRRK2 (Sigma: TRCN0000021459 and TRCN0000021460) or the non-target shRNA control vector (Sigma: SHC002) were transfected into HEK293T cells together with lentivirus packaging plasmids (PLP1, PLP2 and PLP-VSVG, Invitrogen). Culture medium was collected 2 days after transfection and passed through a 0.45-μm nitrocellulose filter. Cells were infected with lentivirus expressing shRNAs and selected with 2 μg ml^−1^ puromycin 2 days post infection.

### Electron microscopy

Wandering third-instar larvae were dissected, prepared and embedded as described in Jia *et al*.^69^. Ultra-thin serial sections of 50 nm thickness were cut from muscle 6, 7 and 12 of hemisegments A2 and A3. Two wild-type *w*^*1118*^ (55 synaptic profiles from type Ib boutons), two *dLrrk*^*e03680*^ larvae (191 synaptic profiles from type Ib boutons) were used for this study. Electron micrographs were taken at a magnification of × 19,500 and × 40,000 for figures and measurements on a Philips/FEI CM120 electron microscope equipped with a digital camera. Serial reconstruction and analysis was conducted on Reconstruct v.1.1.0.0 Software^70^.

### Statistical analysis

Data are presented as mean±s.e.m. (*n*=number of NMJs unless otherwise indicated). Histograms and frequency distributions were generated using Excel software (Microsoft Corporation). For all pair-wise comparisons, a two-tailed Student's *t*-test (Excel) was used to determine statistical significance. In all other cases, statistical significance was determined using PASW 7.0 software (SPSS Inc.). Each data set was first subjected to a variance test. In the absence of a significant difference, one-way analysis of variance followed by Tukey's *post-hoc* test was applied. If there were differences in variance, Games–Howell *post-hoc* test was applied. See Supplementary Table 1 for statistical information related to electrophysiological analysis for each figure.

### Data availability

All data in this manuscript are available from the authors.

## Additional information

**How to cite this article:** Penney, J. *et al*. LRRK2 regulates retrograde synaptic compensation at the *Drosophila* neuromuscular junction. *Nat. Commun.* 7:12188 doi: 10.1038/ncomms12188 (2016).

## Supplementary Material

Supplementary Information Supplementary Figures 1 - 8 and Supplementary Tables 1 - 2
