# Supplementary material for: LRRK2 regulates retrograde synaptic compensation at the Drosophila neuromuscular junction
Source: Nat Commun. 2016 Jul 19;7:12188. doi: 10.1038/ncomms12188 (PMC4960312; doi:10.1038/ncomms12188)
Supplement: Supplementary Information — Supplementary Figures 1 - 8 and Supplementary Tables 1 - 2 [file ncomms12188-s1.pdf]

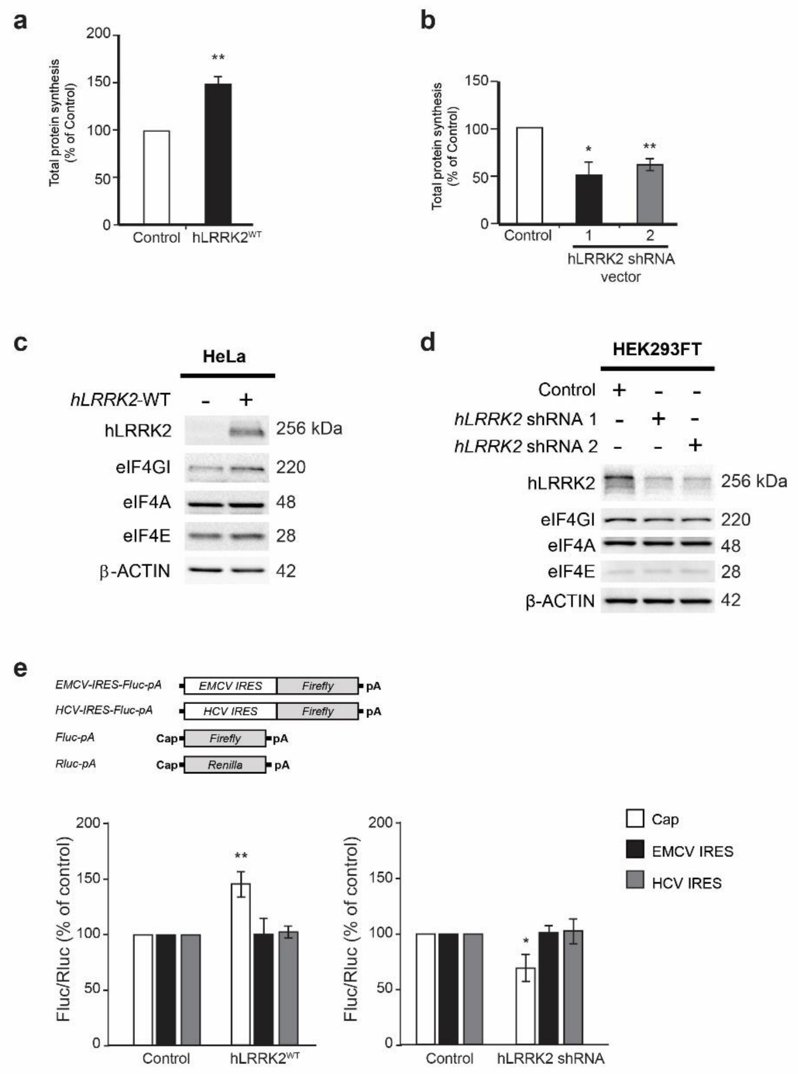

Supplementary Figure 1 | hLRRK2 promotes CAP dependent protein translation.

(a) Overexpression of hLRRK2 in HeLa cells enhances total protein synthesis in [35S] methionine/cysteine incorporation assays. n=3 experiments. **p=0.004, Student's t-test.

(b) Knock down of hLRRK2 using two different shRNAs in Human Embryonic Kidney cells 293FT (HEK293FT) cells reduces total protein synthesis in [35S] methionine/cysteine incorporation assays. n=3 experiments. *p=0.016, **p=0.001, Student's t-test.

(c) Western blot comparing levels of hLRRK2 and cap-binding protein complex components in HeLa cells with or without hLRRK2 overexpression.

(d) Western blot comparing levels of hLRRK2 and cap-binding protein complex components in in HEK293FT cells in control or in response to different shRNAs against hLRRK2.

(e) Overexpression of hLRRK2 promotes cap dependent translation in luciferase reporter assay: 5'-cap dependent Firefly luciferase mRNA reporter and 5'-IRES (Internal Ribosome Entry Site) Firefly luciferase mRNA reporter activity response to hLRRK2-WT overexpression (left) and hLRRK2 shRNA knock down (right).

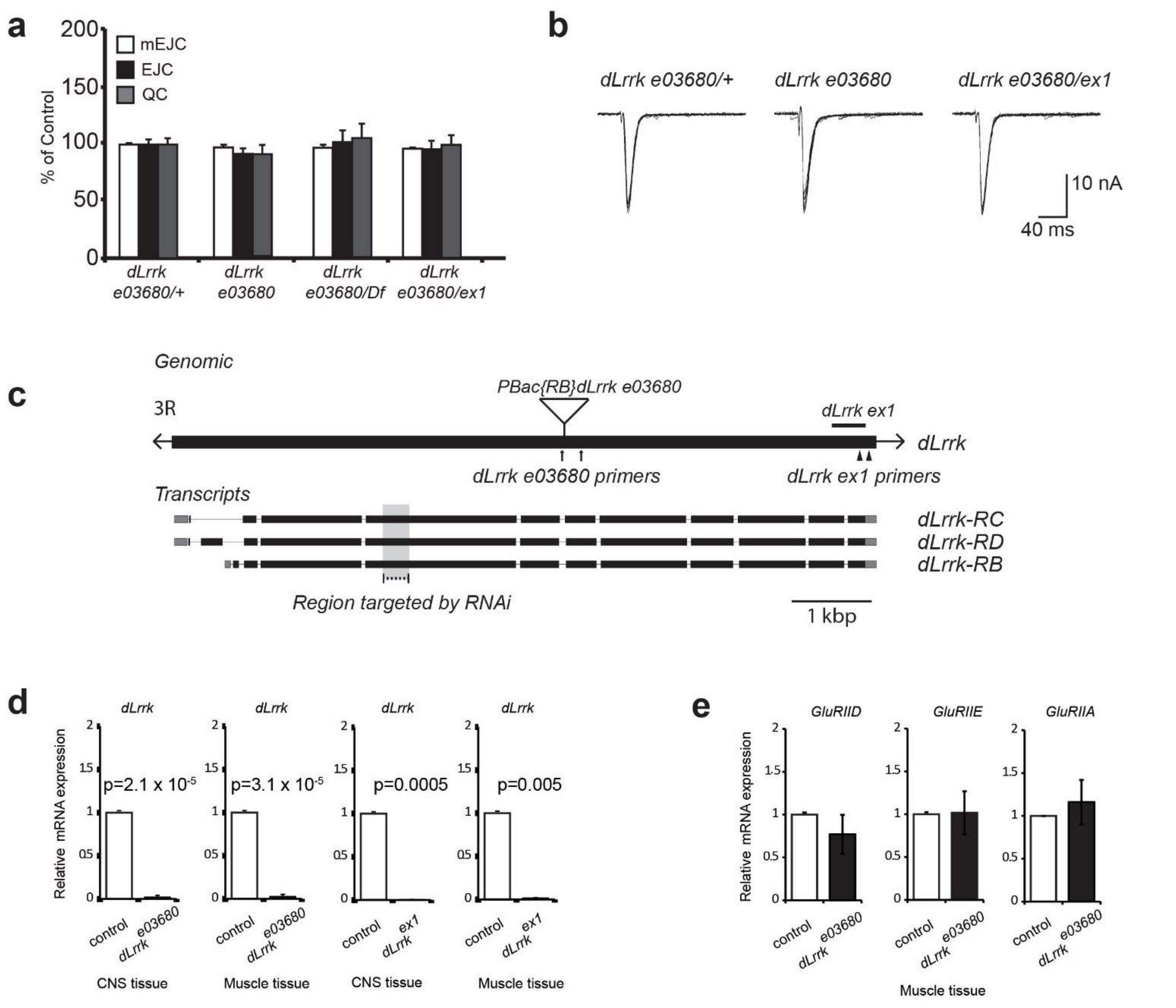

Supplementary Figure 2 | *dLrrk* mutant analysis.

(a) *dLrrk* mutants have normal baseline synaptic transmission. Quantification of mEJC, EJC and QC for *dLrrk* allele combinations as indicated. n= 39, 27, 13 and 23. See also Supplementary Table 1.

(b) Representative EJC traces for three of the genotypes in (a).

(c) Schematic representation of *dLrrk* locus, primer sites and mutants used in this study.

(d) qPCR analysis of *dLrrk* mRNA expression in homozygous *dLrrk*^{e03680} and *dLrrk*^{ex1} mutants. Student's t-test.

(e) qPCR analysis of transcript levels for glutamate receptor subunits *GluRIID* and *GluRIIE* and *GluRIIA* in

dLrrk ^{e03680} mutants. n= 3 experiments.

Error bars represent SEM.

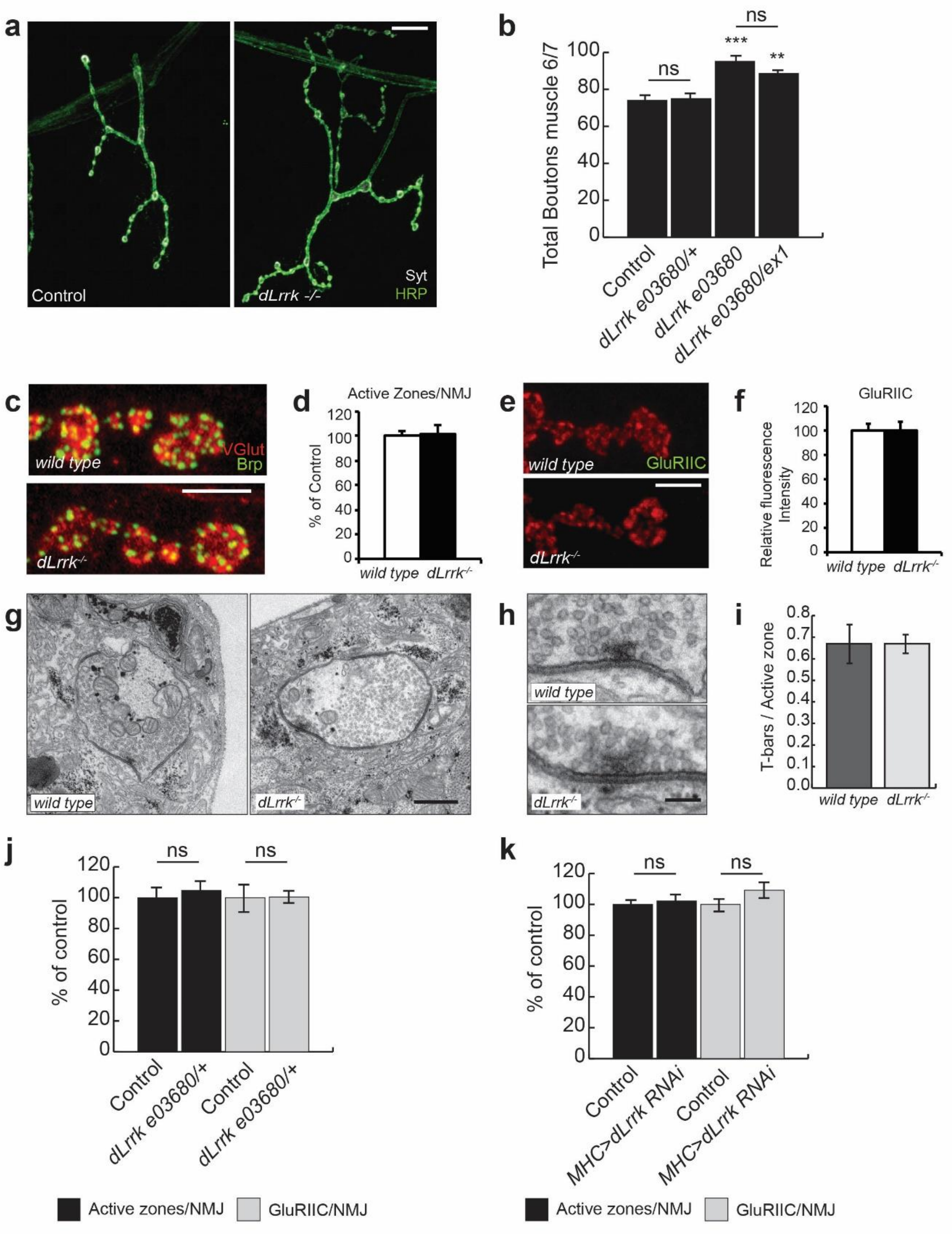

Supplementary Figure 3 | *dLrrk* mutants have a normal number of synaptic release sites.

(a) Muscle 4 NMJs from control (*w1118*) and *dLrrk -/-* (*dLrrk e03680*) larvae stained with anti-synaptotagmin (Syt) and HRP. Scale bar is 10μm.

(b-k) continued on next page

Supplementary Figure 3 | *dLrrk* mutants have a normal number of synaptic release sites.

(b) Quantification of total bouton number on muscle 6/7. N=34, 57, 39, 17 NMJs respectively. ***P<0.001, **P<0.001. Student's t-test. Error bars are SEM.

(c) Terminal boutons from muscle 6/7 NMJs stained with anti-bruchpilot (Brp, green) and anti-VGlut (VGlut, red) in wild type (w^{1118}) and *dLrrk* mutant larvae ($dLrrk^{e03680}$), Scale bar 5 µm.

(d) Quantification of the number of active zones per NMJ from the genotypes shown in (a). n = 10, 10.

(e) Terminal boutons from muscle 4 NMJs of wild type and *dLrrk* mutant larvae, stained with anti-Glutamate receptor subunit IIC (GluRIIC). Scale bar 5 µm.

(f) Quantification of the fluorescent intensity of GluRIIC staining from the genotypes in (c). n = 15 for each.

(g) Electron micrographs (EM) of muscle 6/7 boutons from wild type (*w1118*) and *dLrrk* mutant larvae

($dLrrk^{e03680}$). Scale bar 0.5 µm.

(h) Higher magnification EM of T-bars from wild type (*w1118*) and *dLrrk* mutant larvae ($dLrrk^{e03680}$). Scale bar 0.1 µm.

(i) Quantification of the average number of T-bars per active zone from the genotypes shown in (e, f). n = 55 *w1118* and 191 *dLrrk* synapse profiles. Error Bars are SEM.

(j) Quantification of active zones and GluRIIC number per muscle 4 NMJ expressed as percent of control (*w1118*). No significant difference (ns).

(k) Quantification of active zones and GluRIIC number per muscle 4 NMJ expressed as percent of control (MHC-Gal4/+). n=10, 10 NMJs. No significant difference (ns).

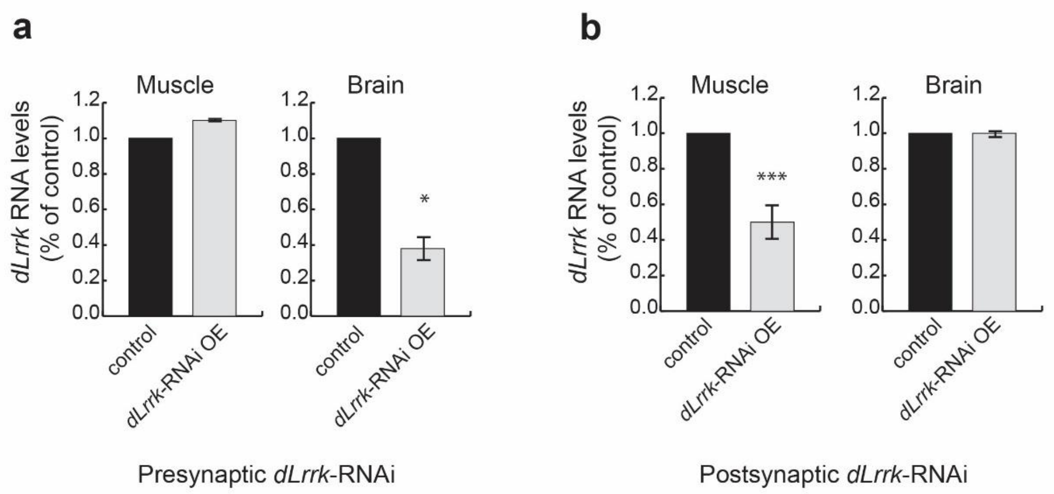

Supplementary Figure 4 | qPCR analysis of *dLrrk* RNAi in neurons and muscles.

(a) Motoneuron RNAi of *dLrrk* reduces *dLrrk* RNA transcript level in the larval brain. Control (*BG380-Gal4*/+), *dLrrk*-RNAi OE (*BG380-Gal4*/+; UAS-*dLRRK-RNAi*/+). n=3.

(b) Muscle RNAi of *dLrrk* reduces *dLrrk* RNA transcript level in larval muscles. Control (*MHC-Gal4*/+) and *dLrrk*-RNAi OE (*MHC-Gal4*/UAS-*dLRRK-RNAi*). n=3. Error bars represent SEM. *p=0.01, ***p=0.0003, Student's t-test.

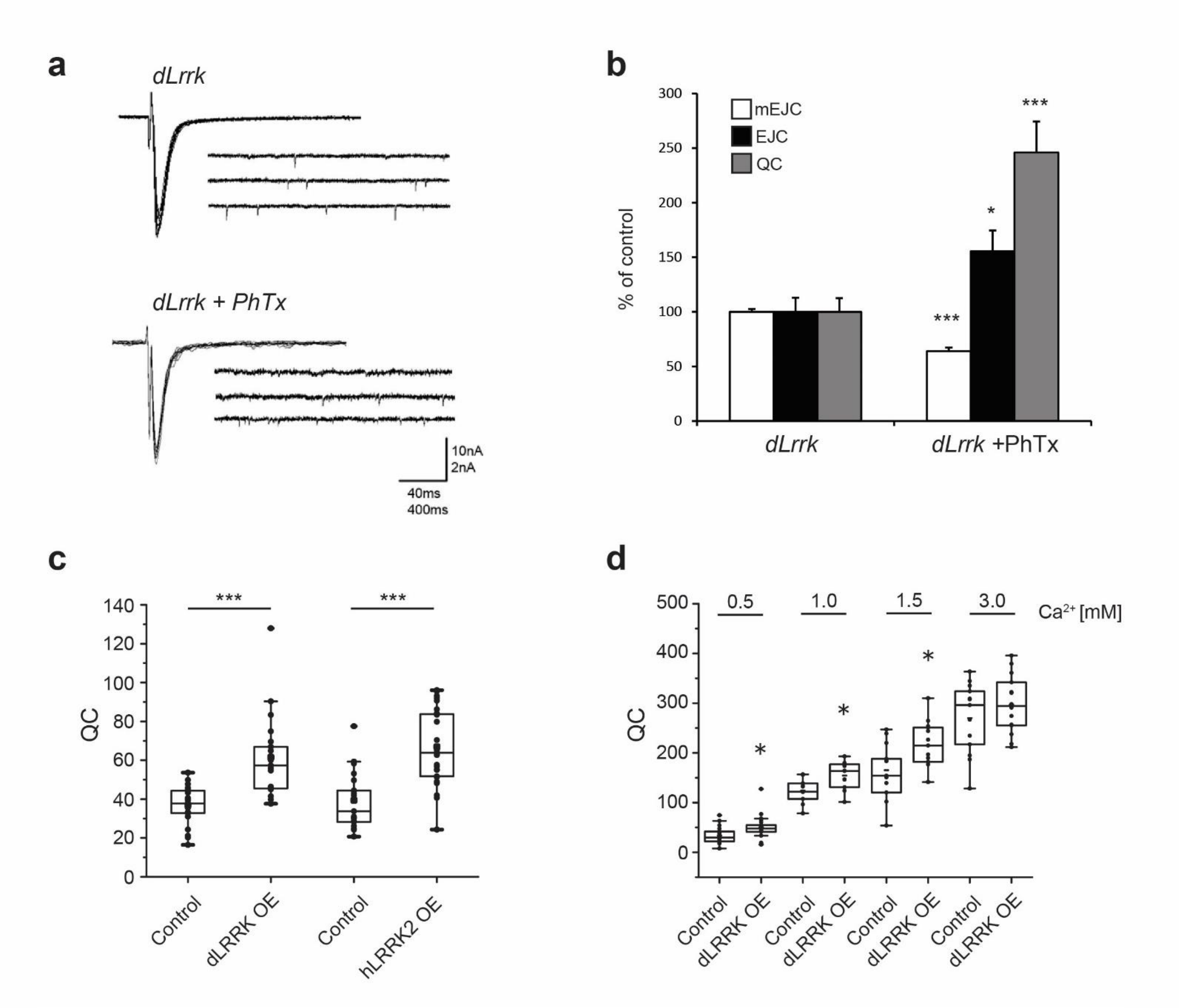

Supplementary Figure 5 | Rapid induction of homeostatic plasticity in *dLrrk* mutants. dLRRK overexpression at different calcium concentrations.

(a) Representative traces of EJCs and mEJCs from *dLrrk* (*dLrrk*^{e03680}) mutants and *dLrrk* mutants treated with PhTx (*dLrrk*^{e03680} + PhTx treatment).

(b) Quantification of mEJC, EJC and QC from *dLrrk* (*dLrrk*^{e03680}) and *dLrrk* + PhTx (*dLrrk*^{e03680} + PhTx treatment). n=8, 8. *p<0.05, ***p<0.001. Student's t-test. Error bars are SEM.

(c) Quantification of QC in control (UAS-*dLRRK*/+), dLRRK OE (UAS-*dLRRK*/*G14-Gal4*), and Control (UAS-*hLRRK2*/+), hLRRK2 OE (*G14-Gal4*/+; UAS-*hLRRK2*/+). n = 21, 20, 20, 28. ***p<0.001, Student's t-test

(d) Quantification of QC in control (*MHC-Gal4*/+) and dLRRK OE (UAS-*dLRRK*/+; *MHC-Gal4*/+) at indicated external calcium concentrations. (control, n=20, 10, 12, 13) and (dLRRK OE, n=20, 11, 13, 15). *p<0.05. Student's t-test for each corresponding pair. Note that at 3mM external calcium the difference in QC is no longer statistically significant. Quantification of the corresponding EJCs is shown in Figure 2c.

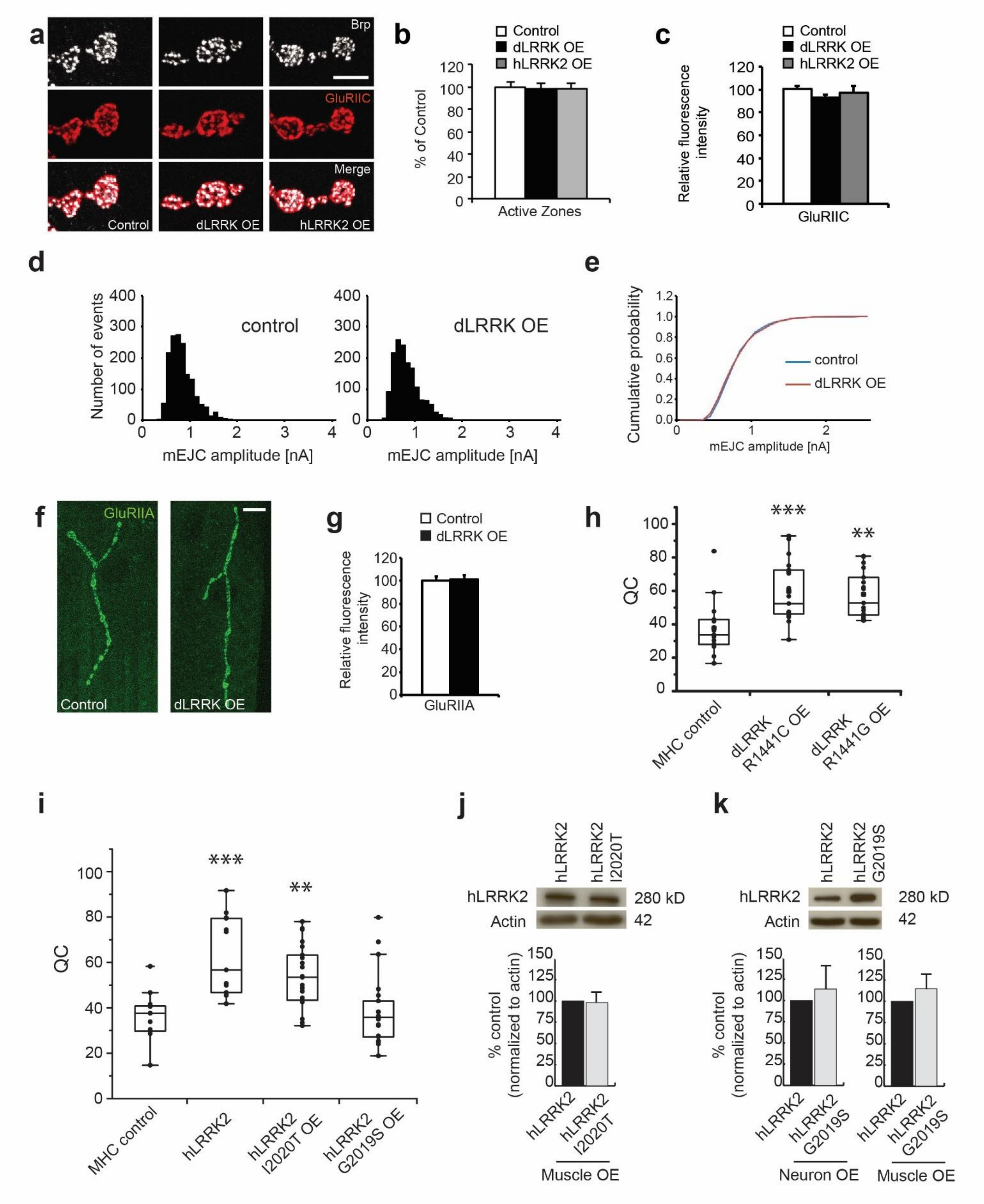

Supplementary Figure 6 | LRRK2 postsynaptic overexpression does not influence synaptic structure.

(a) Terminal boutons from muscle 6/7 NMJs double-stained with anti-Brp (white) and anti-GluRIIC (red) of controls or larvae overexpressing dLRRK or hLRRK2 in the muscle. Scale bar, 5 µm. **(b-k)** Continued on next page.

Supplementary Figure 6 | LRRK2 postsynaptic overexpression does not influence synaptic structure.

(b) Quantification of the number of active zones per NMJ from the genotypes shown in (a). n = 10 for each.

(c) Quantification of the fluorescence intensity of GluRIIC staining from the genotypes shown in (a). n = 14, 14, 10.

(d) mEJC amplitude distribution graphs for control (UAS-*dLRRK*/+) and dLRRK muscle overexpression (OE) larvae (*G14-Gal4*/UAS-*dLRRK*).

(e) Cumulative probability of mEJC amplitude. There is no significant difference in mEJC amplitude distribution between control and dLRRK OE. n=8, 10 (p=0.148 calculated by non-parametric Kolmogorov-Smirnov test).

(f) Muscle 4 NMJs in control (*MHC-Gal4*/+) and dLRRK overexpression in muscle (+/UAS-*dLRRK; MHC-Gal4*/+). Scale bar, 10 µm.

(g) Quantification of the fluorescent intensity of GluRIIA staining from the genotypes shown in (f). n = 16 for each.

(h) Box plots of Quantal Content (QC) for MHC control (*MHC-Gal4/+*), dLRRK R1441C OE (UAS-*dLRRK R1441C*/+; *MHC-Gal4*/+) and dLRRK R1441G OE (*MHC-Gal4*/UAS-*LRRK R1441G*). n=15, 18, 18. **p<0.01, *** p<0.001.

(i) Box plots of Quantal Content (QC) for MHC control (*MHC-Gal4*/+), hLRRK2 (*MHC-Gal4/*UAS-*hLRRK2*), hLRRK2 I2020T OE (*MHC-Gal4*/UAS-*hLRRK2 I2020T*) and hLRRK2 G2019S OE (*MHC-Gal4*/UAS-*hLRRK2 G2019S*). n=12, 13, 20, 20. **p<0.01, ***p<0.001.

(j) Western blot analysis of hLRRK2 I2020T expression compared to hLRRK2 expression from larval muscle extracts. Representative western blot with actin control (top), quantification of hLRRK2 (*MHC-Gal4/*UAS-*hLRRK2*) and hLRRK2 I2020T (*MHC-Gal4*/UAS-*hLRRK2 I2020T*) expression normalized to actin and expressed as a percentage of control (bottom). n=3 experiments.

(k) Western blot analysis of hLRRK2 G2019S expression compared to hLRRK2 expression from larval tissue. Representative western blot of brain expressed hLRRK2. Actin serves as loading control (top). Quantification of hLRRK2 expression from larval brain tissue from motoneuron driven hLRRK2 (*OK6-Gal4*/+; UAS-*hLRRK2*/+) and hLRRK2 G2019S (*OK6-Gal4*/+; UAS-*hLRRK2 G2019S*/+) (bottom left) and muscle tissue from muscle driven hLRRK2 (UAS-*hLRRK2*/*MHC-Gal4*) and hLRRK2 G2019S (UAS-*hLRRK2 G2019S*/*MHC-Gal4*) (bottom right). Quantifications normalized to actin and expressed as a percentage of control. n=3 experiments.

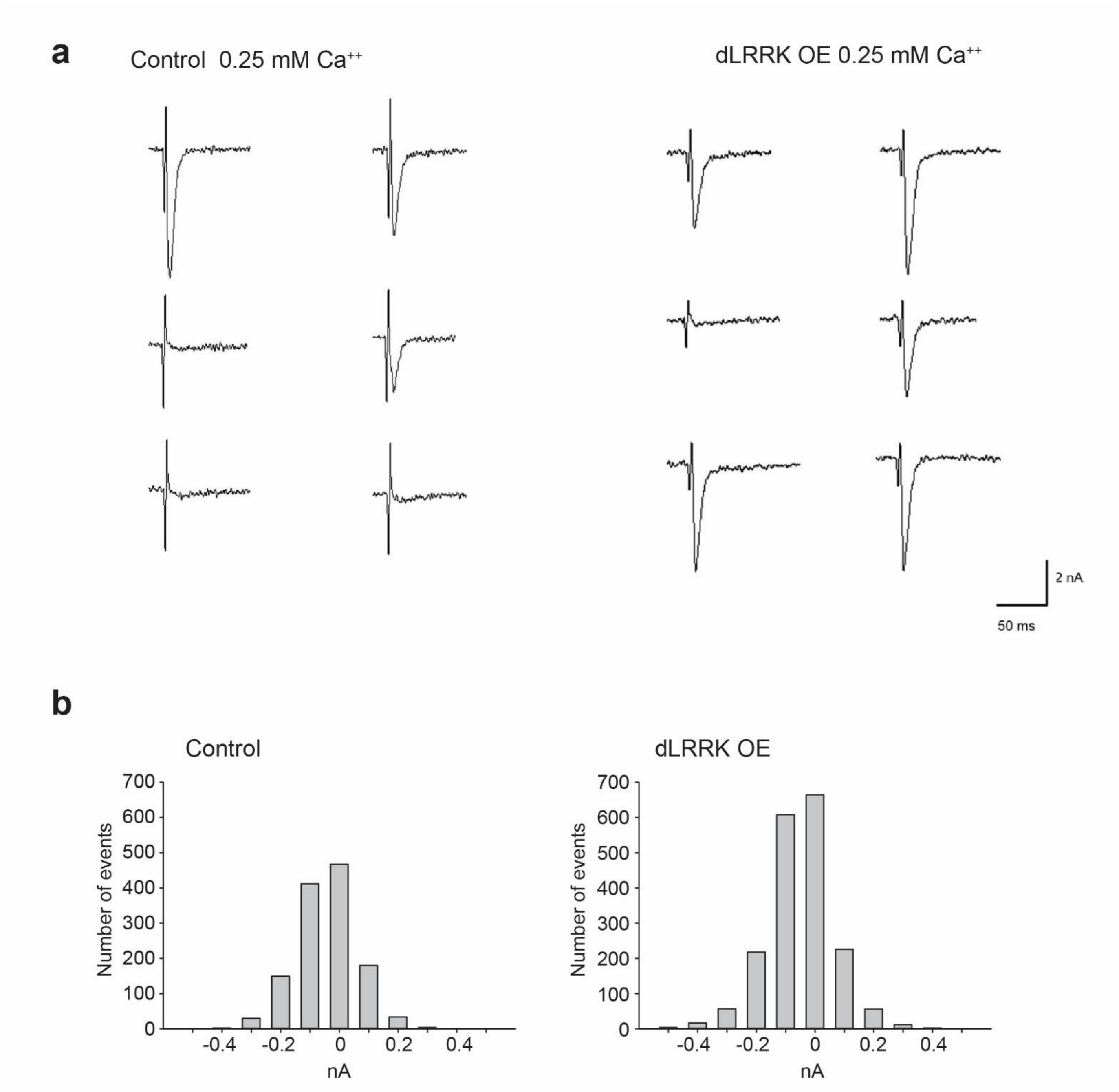

Supplementary Figure 7 | Representative traces and measurement of baseline noise for failure analysis

(a) Representative sample traces of consecutive stimulation used for failure analysis shown in Figure 3a. Control (*G14-Gal4*/+) and dLRRK OE (*G14-Gal4*/*UAS-dLRRK*).

(b) Distribution of baseline noise measured for failure analysis in Figure 3a. Control (*G14-Gal4*/+) and dLRRK OE (*G14-Gal4*/*UAS-dLRRK*). n=7, 8 NMJs.

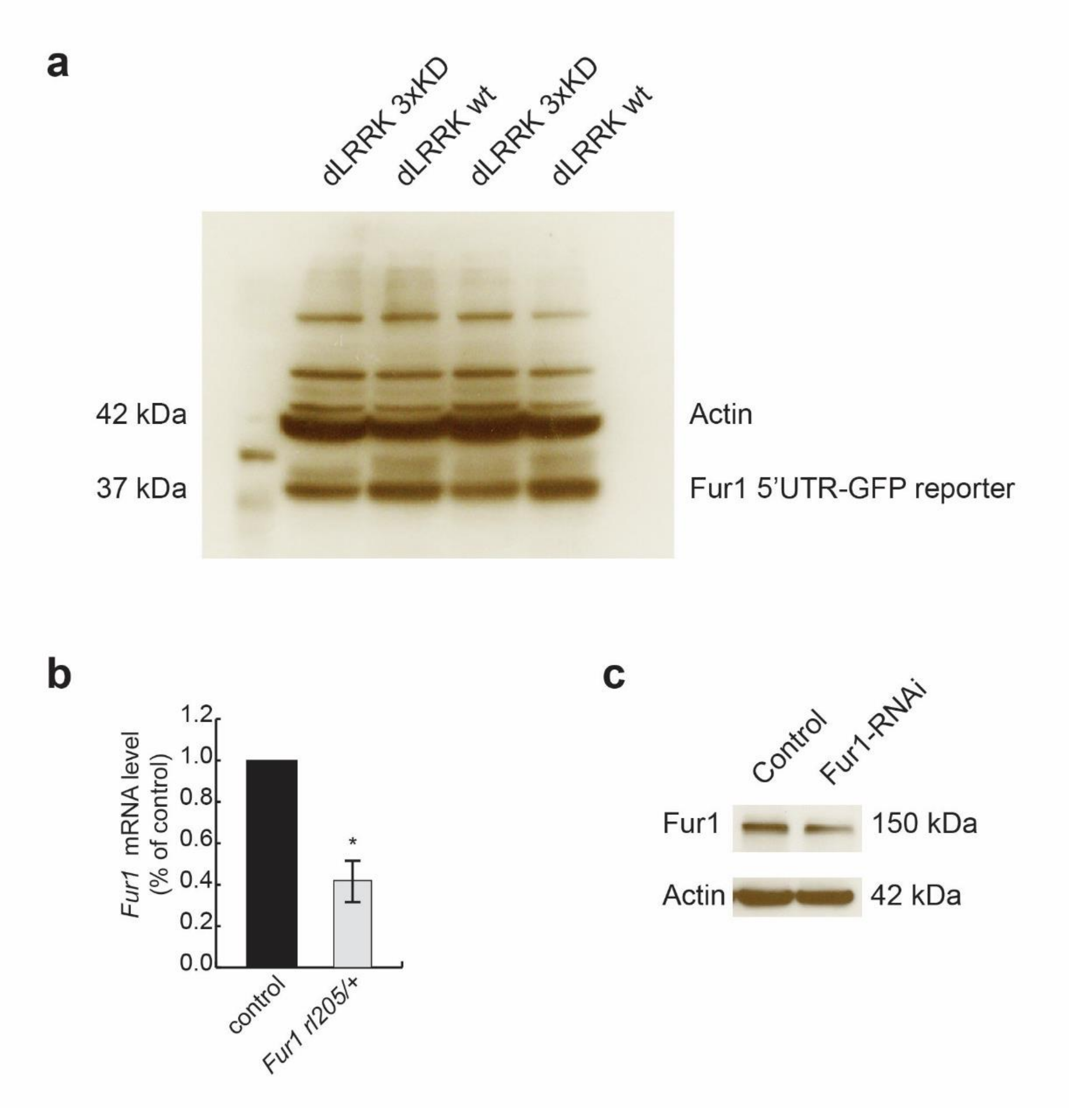

Supplementary Figure 8 | Furin1 reporter response to dLRRK overexpression and Fur1 level controls in heterozygous and knockdown larvae

(a) Uncropped western blot from Figure 5b of *in vivo* Fur1-5'UTR-eGFP reporter expression when co-expressed with either dLRRK^{3KD} (+/UAS-*Fur1*-5'UTR-*eGFP*; UAS-*dLRRK*^{3KD}/*MHC-Gal4*) or wild-type dLRRK^{wt} (UAS-*dLRRK*^{wt}/UAS-*Fur1*-5'UTR-*eGFP*; +/*MHC-Gal4*).

(b) Quantitative PCR analysis of *Furin1* transcript levels in *Fur1*^{rl205} heterozygous flies. n=4 experiments. Error bars represent SEM. *p=0.029 Student's t-test.

(c) Representative western blot of Furin1 levels in control (*MHC-Gal4/*+) and *Fur1*-RNAi (*MHC-Gal4/*UAS-*Fur1-RNAi*) larvae. n=3 experiments.

Supplementary Table 1

Electrophysiology data

Tukey (T) or Games-Howell (GH) post-hoc test was applied after one-way ANOVA. T test was applied for all pair-wise comparison.

Data for Figure 1b

Genotype	mEJC (T)	EJC (T)	QC (T)	N
MHC-Gal4/+	0.682±0.0156	24.043±2.065	34.863±2.462	21
dLrrk^{e03680}/+	0.679±0.105	23.39±1.599	34.475±2.315	27
GluRIIA^{MR}/+; *MHC-Gal4*/+	0.431±0.0123 (p<0.001)	23.709±1.456	55.563±3.83 (p<0.0001)	19
GluRIIA^{MR}; *MHC-Gal4*/*dLrrk*^{e03680}	0.424±0.009 (p<0.0001)	17.383±1.505 (p=0.0042)	41.071±3.601 (p=0.125)	19

Data for Figure 1c

Genotype	mEJC (T)	EJC (GH)	QC (GH)	N
w1118	0.742±0.0126	25.280±1.34	34.23±1.809	32
GluRIIA$^{-/-}$	0.451±0.0155 (p<0.0001***)	22.393±2.112 (p=0.4035)	50.433±4.915 (p<0.05)	20
GluRIIA$^{-/-}$; *dLrrk* $^{-/-}$	0.426±0.0124 (p<0.0001***)	13.711±1.442 (p<0.0001***)	31.966±3.170	16

Data for Figure 1e

Genotype	mEJC (T)	EJC (T)	QC (T)	N
24B-Gal4/+	0.721±0.0131	24.347±1.917	33.900±2.622	19
24B-Gal4/*UAS-dLRRK-RNAi*	0.735±0.0161	22.993±1.969	30.838±2.831	20
GluRIIA$^{-/-}$;24B-Gal4/+	0.424±0.0096 (p<0.0001***)	22.786±1.582 (p=0.806)	53.877±3.713 (p=0.0007***)	18
GluRIIA$^{-/-}$;*24B-Gal4 /UAS-dLRRK-RNAi*	0.433±0.0121 (p<0.0001***)	12.435±1.722 (p<0.0001***)	29.124±4.252 (p=0.612)	19

Data for Figure 1f

Genotype	mEJC (T)	EJC (T)	QC (T)	N
BG380/+;GluRIIA^{-}/+ *UAS-Dcr-2/+*	0.654±0.0094	18.375±2.598	28.374±4.037	17
BG380/+; GluRIIA$^{-/-}$; UAS-*Dcr-2*/+	0.411±0.0119 (p<0.0001)	17.464±2.045	41.851±4.514 (p=0.027)	18
BG380/+; GluRIIA$^{-/-}$;*UAS-dLRRK-RNAi/UAS-Dcr-2*	0.436±0.0125 (p<0.0001)	18.954±2.157	43.341±4.867 (p=0.019)	20

Data for Figure 2b and Supplementary 5c

Genotype	mEJC (T)	EJC (T)	QC (T)	N
UAS-dLRRK/+	0.731±0.0109	26.867±1.613	36.848±2.190	21
UAS-dLRRK/+; +/*G14-Gal4*	0.715±0.0211 (p=0.504)	44.203±3.670 (p<0.0001***)	61.503±4.744 (p<0.0001***)	20
UAS-hLRRK2/+	0.686±0.0132	26.659±2.029	39.448±3.261	20
UAS-hLRRK2/+; +/*G14-Gal4*	0.684±0.0110 (p=0.919)	45.778±2.472 (p<0.0001***)	66.940±3.589 (p<0.0001***)	28

Data for Figure 2c and Supplementary 5d

Genotype	EJC (Ca++ [mM])				N
	(0.5)	(1.0)	(1.5)	(3.0)	
MHC-GAL4/+	24.0607±2.715	86.038±5.804	115.812±10.89	162.775±9.0319	20, 10, 12, 13
UAS-dLRRK/+; MHC-GAL4/+	36.3037±3.218 (p=0.00605)	110.875±4.781 (p=0.00354)	150.2176±9.8245 (p=0.0276)	190.352±6.8875 (p=0.0207)	20, 11, 13, 15

Data for Figure 2f and Supplementary Figure 6h

Genotype	mEJC (T)	EJC (T)	QC (T)	N
MHC-GAL4/+	0.683±0.022	25.807±2.914	11.337±4.282	15
UAS-dLRRK R1441C/+; MHC-GAL4/+	0.698±0.015	41.463±2.761 (p=1.46x10^{-4})	59.823±4.259 (p=8.4x10^{-4})	18
MHC-GAL4/UAS-dLRRK R1441G	0.709±0.0203	40.286±1.547 (p=4.3x10^{-4})	57.987±3.193 (p=0.00225)	18

Data for Figure 2g and Supplementary Figure 6i

Genotype	mEJC (T)	EJC (T)	QC (T)	N
MHC-GAL4/+	0.694±0.0143	25.445±2.116	36.698±3.1	12
MHC-GAL4/UAS-hLRRK2	0.709±0.0253	40.594±2.739	62.966±4.821	13
MHC-GAL4/UAS-hLRRK2 I2020T	0.703±0.0203	38.074±2.167	54.531±3.129	20
MHC-GAL4/UAS-hLRRK2 G2019S	0.624±0.0112	24.359±2.223	39.0745±3.527	20

Data for Figure 3a, Supplementary 7a, b.

Genotype	mEJC #events	Failures	Total (EJCs + failures)	# events for noise analysis	QC	n
G14-Gal4/+	1784	409	2572	1277	1.9539±0.209	7
UAS-dLRRK/*G14-Gal4*	1828	152	3024	1862	3.3131±0.317<br>(p=0.00416)	8

Data for Figure 3d

Genotype	Number of Release Ready Vesicles (N)	n
MHC-Gal4/+	312.238±50.379	5
UAS-dLRRK/+; *MHC-Gal4*/+	532.79±72.948<br>(p=0.0462)	7

Data for Figure 3e

Genotype	Pvr	N
MHC-Gal4/+	0.099917±0.0163	5
UAS-dLRRK/+; *MHC-Gal4*/+	0.103598±0.0196	7

Data for Figure 3g

Genotype	RRP	N
MHC-Gal4/+	1107.43±97.58	8
UAS-dLRRK/+; *MHC-Gal4*/+	1531.831±107.94<br>(p=0.01169)	10

Data for Figure 4b

Genotype	mEJC (T)	EJC (T)	QC (T)	N
G14-Gal4/+	0.747±0.0175	21.640±1.976	28.947±2.578	13
G14-Gal4/*UAS-dLRRK*	0.695±0.0183 (p=0.231)	39.431±3.949 (p=0.0006***)	56.481±5.311 (p<0.0001***)	18
G14-Gal4/*UAS-dLRRK*;*eIF4E*/+	0.736±0.0171 (p=0.993)	27.066±1.663 (p=0.706)	36.889±2.235 (p=0.658)	16
G14-Gal4/+; +/*UAS-hLRRK2*	0.725±0.0166 (p=0.937)	49.331±3.547 (p<0.0001***)	68.442±5.389 (p<0.0001***)	10
G14-Gal4/+; *eIF4E*/*UAS-hLRRK2*	0.696±0.0201 (p=0.385)	32.893±3.084 (p=0.143)	47.622±4.473 (p=0.0478*)	10

Data Figure 4c

Genotype	mEJC (GH)	EJC (GH)	QC (T)	N
UAS-*dLRRK*/+	0.731±0.0109	26.867±1.613	36.848±2.190	21
UAS-*dLRRK*/G14-Gal4	0.715±0.0211 (p=0.779)	44.203±3.670 (p=0.0006***)	61.503±4.744 (p<0.0001***)	20
UAS-*dLRRK*/G14-Gal4;+/*S6k*	0.750±0.0155 (p=0.571)	24.870±2.267 (p=0.755)	32.838±2.717 (p=0.677)	20
Genotype	mEJC (T)	EJC (T)	QC (T)	N
UAS-*hLRRK*2/+	0.686±0.0132	26.659±2.029	39.448±3.261	20
+/G14-Gal4; UAS-*hLRRK*2/+	0.684±0.0110 (p=0.995)	45.778±2.472 (p<0.0001***)	66.940±3.590 (p<0.0001***)	28
+/G14-Gal4;UAS-*hLRRK*2/*S6k*	0.679±0.0172 (p=0.942)	28.338±2.338 (p=0.885)	41.786±3.283 (p=0.896)	20

Data Figure 4d

Genotype	mEJC	EJC	QC	N
UAS-*dLRRK*/+; MHC-Gal4/+	0.689±0.025	34.519±2.72	55.500±5.07	14
UAS-*dLRRK*/TorP; MHC-Gal4/+	0.689±0.019	25.159±2.696 (p=0.0409)	39.084±4.343 (p=0.0411)	19

Data Figure 4e

Genotype	mEJC	EJC	QC	N
MHC-Gal4/UAS-Tor	0.737±0.025	39.599±3.32	54.844±5.009	16
MHC-Gal4/UAS-*Tor, dLrrk e03680*	0.727±0.0238	30.224±2.817 (p=0.0231)	41.580±3.535 (p=0.0197)	19

Data for Figure 4f

Genotype	mEJC (T)	EJC (T)	QC (T)	N
MHC-Gal4/+	0.716±0.0184	21.526±2.809	29.751±3.672	10
MHC-Gal4/+ with CHX	0.735±0.0222 (p=0.949)	24.403±1.933 (p=0.908)	33.775±3.186 (p=0.905)	10
+/*UAS-dLRRK*; *MHC-Gal4*/+	0.733±0.0193 (p=0.971)	36.295±2.140 (p=0.0006***)	49.606±3.001 (p=0.0009***)	10
+/*UAS-dLRRK*; *MHC-Gal4*/+ with CHX	0.761±0.0214 (p=0.510)	23.523±3.147 (p=0.979)	30.883±4.045 (p=0.999)	8
+/*UAS-dLRRK*; *MHC-Gal4*/+ with RAPA	0.715±0.0100 (p=0.999)	18.562±2.156 (p=0.917)	26.003±2.977 (p=0.939)	8

Data for Figure 6a

Genotype	mEJC (T)	EJC (T)	QC (T)	N
MHC-Gal4/+	0.724±0.0195	28.824±3.02	37.816±4.39	10
+/UAS-*dLRRK*; MHC-Gal4/+	0.727±0.0253	59.726±4.400 (p=0.000121)	79.336±5.54 (p=0.000114)	10
+/UAS-*dLRRK*; MHC-Gal4/$Furin1^{rl205}$	0.719±0.019	30.756±5.626 (p=0.000273)	44.858±7.59 (p=0.00104)	10

Data for Figure 6c

Genotype	mEJC (T)	EJC (GH)	QC (GH)	N
MHC-Gal4/+	0.748±0.020	25.134±2.153	33.958±3.049	18
+/UAS-*dLRRK*; MHC-Gal4/UAS-*eGFP*	0.761±0.016 (p=0.892)	40.960±3.874 (p<0.05)	53.41±5.41 (p=0.003)	18
+/UAS-*dLRRK*; MHC-Gal4/UAS-*Fur1*-RNAi	0.751±0.0240	23.333±2.168	31.458±3.1	18

Data for Figure 7b

Genotype	mEJC (T)	EJC (T)	QC (T)	N
MHC-Gal4/+	0.712±0.0257	24.912±1.993	35.461±3.038	20
+/UAS-dLRRK; MHC-Gal4/UAS-$hLRRK2^{WT}$	0.710±0.0204 (p=0.996)	47.008±2.736 (p<0.0001***)	67.100±4.395 (p<0.0001***)	20
+/UAS-dLRRK; MHC-Gal4/UAS-$hLRRK2^{G2019S}$	0.656±0.0153 (p=0.132)	23.627±2.995 (p=0.936)	36.301±4.433 (p=0.988)	22

Data for Figure 7d

Genotype	mEJC (T)	EJC (T)	QC (T)	N
GluRIIA$^{-/-}$	0.447±0.0102	28.161±1.932	63.824±4.830	20
GluRIIA$^{-/-}$;24B-Gal4/UAS-hLRRK2^{WT}	0.494±0.0149 (p=0.0508)	30.525±1.895 (p=0.672)	62.804±4.012 (p=0.986)	20
GluRIIA$^{-/-}$;24B-Gal4/UAS-hLRRK2^{G2019S}	0.465±0.0153 (p=0.624)	18.207±1.944 (p=0.00147**)	39.251±4.371 (p=0.0006***)	22

Data referred to in Text:

Genotype	mEJC (T)	EJC (T)	QC (T)	N
24B-Gal4/+	0.739±0.0207	19.857±1.974	19.857±1.974	21
24B-Gal4/*UAS-hLRRK2*	0.712±0.0120 (p=0.495)	36.156±2.204 (p<0.0001***)	51.197±3.326 (p<0.0001***)	19

Data for Supplementary Figure 2a

Genotype	mEJC (T)	EJC (T)	QC (T)	N
dLrrk^{e03680}/+	0.706±0.012	23.488±1.156	33.538±1.730	39
dLrrk^{e03680}	0.698±0.0152	21.240±1.440	30.064±2.252	27
dLrrk^{e03680} /*Df*	0.689±0.0116	23.846±2.595	35.076±4.266	13
dLrrk^{e03680} /*dLrrk*^{ex1}	0.679±0.0.0095 (P=0.251)	22.418±1.60 (P=0.641	33.334±2.479 (P=0.561)	23

Data for Supplementary Figure 5b

Genotype	mEJC (T)	EJC (T)	QC (T)	N
dLrrk e03680	0.7434±0.0189	18.702±2.413	25.0625±3.154	8
dLrrk e03680 + PhTx	0.4746±0.0252 (p=1.09x10^{-6})	29.0799±3.558 (p=0.03268)	61.641±7.121 (p=0.000846)	8

Supplementary Table 2

5'UTR Luciferase reporter assay identifies *Fur1* as an hLRRK2 responsive gene

	Gene-Transcript	Length (nt)	ΔG (kcal/mol at 25°C)	5'UTR luciferase reporter activity (% of control)	Fold change in luciferase activity with hLRRK2 WT. (Normalized to PS2 control)
1	Akt1-RA	732	-235.008	22.4	1.09
2	Antp-RH	1730	-499.902	1.3	0.98
3	Axn-RA	1454	-452.489	5.2	1.16
4	CASK-RA	1415	-391.592	0.5	0.97
5	CG15737-RA	556	-179.89	33.2	1.11
6	CG4069-RA	88	-15.74	62.7	0.98
7	CG4213-RB	391	-164.379	48.7	1.02
8	CG8701-RA	179	-52.354	88.3	0.91
9	ci-RA	1173	-340.805	7.5	1.11
10	dock-RA	1189	-439.434	9.1	0.97
11	Dscam-RBG	519	-182.183	40.5	1.12
12	fur1-RD	735	-221	0.7	1.42
13	gbb-RA	172	-44.466	77.4	0.98
14	GluRIIA-RI	230	-75.711	24.5	0.95
15	GluRIIB-RI	109	-36.031	75.2	0.90
17	InR-RB	1122	-329.732	2.46	0.90
18	Klp61F-RA	295	-98.224	54.62	0.96
19	Med-RB	616	-212.819	14.1	0.98
20	nmo-RB	1051	-281.911	4.5	1.28
21	numb-RA	793	-236.676	0.3	0.94
22	orb2-RB	578	-186.892	25.6	1.09
23	pasha-RA	561	-183.432	8.68	0.92
24	Ptp10D-RG	693	-185.57	97.2	0.98
25	s6k-RA	557	-198.378	12.6	0.91
26	sli-RA	1157	-409.985	17	1.11
27	SP2637-RB	356	-98.475	37.7	1.00
28	Spn4-RL	636	-201.288	5.26	0.90
29	tkv-RD	989	-325.531	11.3	1.05
30	unc-115b-RE	1168	-456.3	0.59	1.10
31	unc-5-RA	648	-242.373	27.9	1.02
32	vglut-RA	627	-195.1	31.1	1.15
33	CG14535-RA	297	-90.025	74.36	0.74
34	CG33970-RA	631	-199.067	94.07	0.66
35	CG8090-RA	170	-45.616	33.5	0.71
36	Egr-RA	646	-203.87	25.8	0.79
37	GlcAT-P-RB	1012	-295.329	9.4	0.75
38	PLC21C-RC	740	-231.388	25.4	0.76
